# Site-Specific Vesicular Drug Delivery System for Skin Cancer: A Novel Approach for Targeting

**DOI:** 10.3390/gels7040218

**Published:** 2021-11-16

**Authors:** Manisha Pandey, Hira Choudhury, Bapi Gorain, Shao Qin Tiong, Grace Yee Seen Wong, Kai Xin Chan, Xuan They, Wei Shen Chieu

**Affiliations:** 1 Department of Pharmaceutical Technology, School of Pharmacy, International Medical University, Bukit Jalil, Kuala Lumpur 57000, Malaysia; 2 Department of Pharmaceutical Sciences and Technology, Birla Institute of Technology, Mesra, Ranchi 835215, India; bapi.gorain@taylors.edu.my; 3 Center for Drug Delivery and Molecular Pharmacology, Faculty of Health and Medical Sciences, Taylor’s University, Subang Jaya 47500, Malaysia; 4 Undergraduate, School of Pharmacy, International Medical University, Bukit Jalil, Kuala Lumpur 57000, Malaysia; TIONG.SHAOQIN@student.imu.edu.my (S.Q.T.); gracewong.yeeseen@student.imu.edu.my (G.Y.S.W.); chan.kaixin@student.imu.edu.my (K.X.C.); they.xuan@student.imu.edu.my (X.T.); chieu.weishen@student.imu.edu.my (W.S.C.)

**Keywords:** skin cancer, vesicular drug delivery system, nanogels

## Abstract

Skin cancer, one of the most prevalent cancers worldwide, has demonstrated an alarming increase in prevalence and mortality. Hence, it is a public health issue and a high burden of disease, contributing to the economic burden in its treatment. There are multiple treatment options available for skin cancer, ranging from chemotherapy to surgery. However, these conventional treatment modalities possess several limitations, urging the need for the development of an effective and safe treatment for skin cancer that could provide targeted drug delivery and site-specific tumor penetration and minimize unwanted systemic toxicity. Therefore, it is vital to understand the critical biological barriers involved in skin cancer therapeutics for the optimal development of the formulations. Various nanocarriers for targeted delivery of chemotherapeutic drugs have been developed and extensively studied to overcome the limitations faced by topical conventional dosage forms. A site-specific vesicular drug delivery system appears to be an attractive strategy in topical drug delivery for the treatment of skin malignancies. In this review, vesicular drug delivery systems, including liposomes, niosomes, ethosomes, and transfersomes in developing novel drug delivery for skin cancer therapeutics, are discussed. Firstly, the prevalence statistics, current treatments, and limitations of convention dosage form for skin cancer treatment are discussed. Then, the common type of nanocarriers involved in the research for skin cancer treatment are summarized. Lastly, the utilization of vesicular drug delivery systems in delivering chemotherapeutics is reviewed and discussed, along with their beneficial aspects over other nanocarriers, safety concerns, and clinical aspects against skin cancer treatment.

## 1. Introduction

### 1.1. Skin Cancer and Skin Cancer Prevalence Statistics

Skin is known as the most prominent human body organ with approximately 1.8 m^2^ surface area. Skin mainly serves as a protective barrier against ultraviolet (UV) radiation, mechanical injury, chemicals, and microorganisms [1,2]. However, when the cells within the skin epidermis undergo neoplastic changes, this results in skin cancer [1]. Globally, skin cancer is one of the most common types of cancer, especially among Caucasians [2,3,4]. The Caucasian population has a relative lack of skin pigmentation than the dark-skinned population [5]. Australia is the nation with the highest skin-cancer incidence globally [3]. In Malaysia, skin cancer is the tenth most common cancer, based on the third report of the National Cancer Registry, Malaysia (2003–2005) [6]. Based on GLOBOCAN 2020 [7], the new incidence of melanoma of skin melanoma is ranked 26^th^ among 35 types of cancer in Malaysia. Hence, skin cancer is a public health issue and contributes to a rise in the economic burden in its treatment [8]. However, skin cancer is preventable, since chronic UV exposure is the most significant risk factor [3,4,9]. Notably, the patterns of sunlight exposure are also associated with the types and risks of skin cancer [4]. Therefore, prevention strategies, for instance, minimizing sun exposure and using a broad-spectrum sunscreen, should be practiced in daily life [4,9].

Skin cancer is differentiated based on the type of skin cells involved in the tumor mass. Malignant melanoma (MM) and non-melanoma skin cancer (NMSC) are the two broad classifications of skin cancer [10]. Squamous cell carcinoma (SCC) and basal cell carcinoma (BCC) are both the most frequently diagnosed NMSC, making up 99% of all NMSCs [11,12]. Globally, there are 2 million to 3 million cases of NMSC that occur each year [5]. According to GLOBOCAN 2020 [13], the number of new cases and deaths of NMSC worldwide are 1,198,073 and 63,731, respectively. There was an increase in the incidence of NMSC in comparison to 2018. Among 36 cancers types, NMSC has ranked the fifth most prevalent cancer worldwide based on GLOBOCAN 2018 [3,14]. Australia and New Zealand have remained as the countries with the highest incidence of NMSC [3,13]. NMSC has a lower potential of invasion and is less aggressive; hence, it has better survival rates and prognoses than melanoma skin cancer (MSC) [15]. The less common NMSCs include primary cutaneous B-cell lymphoma, dermatofibrosarcoma protuberans, Kaposi sarcoma, and Merkel cell carcinoma [11].

BCC, the most prevalent form of skin cancer, comprises 80 to 85% of all cases of NMSC [4,9]. It originates from the skin cells in the stratum basale and its appendages [11,12]. The tumor of BCC is generally slow-growing and causes local invasion [11]. However, it rarely metastasized to the distant body region [1,11]. UV light exposure, especially, UVB light is the most frequent cause of BCC [11]. There is also a significant association between intermittent intense sunlight exposure and BCC development through UV-driven mutagenesis, in contrast to the risk of SCC, which is strongly related to chronic sunlight exposure [4,16,17]. The mutations of the *p53* tumor suppressor gene induced by UV light would result in loss of inhibiting cell division [11]. Since UV radiation triggers the cell mutation, BCC is more commonly found on sun-exposed skin areas, for instance, ears, backs of hands, face, and nose [1]. For the clinical presentation, nodular BCC is the most prevalent subtype, accounting for about 60% of cases. Nodular BCC is appeared as a pearly, shiny, and rolled border papule along with telangiectasias and may be ulcerated [1,16]. There is an increasing incidence of BCC with age, whereby BCC is more frequently occurs in the elderly population due to cumulative exposure to sunlight and other exogenous damage [17]. Moreover, SCC, the second most prevalent form of skin cancer, makes up 15 to 20% of all NMSC cases [4,12]. SCC develops from the uncontrolled proliferation of squamous keratinocytes and metastatic [1]. At the early stage of SCC, the tumor is confined to the epidermis only, known as Bowen disease [12]. When SCC becomes more invasive, it can invade the dermis and even hypodermis, at which it has the potential to metastasize [12]. In addition, SCC could arise from actin keratosis, the precancerous lesion to SCC [11]. SCC is mainly caused by UV radiation affecting individuals with Type I or II skin types [1]. The clinical presentation of SCC includes well-demarcated borders and crusting of rough and red elevations of the skin [9]. In short, NMSC is usually curable, by which both BCC and SCC could be managed in outpatient settings while immediate treatment is needed to reduce local invasion in SCC [9,11,12].

Apart from skin cells in stratum basale and squamous keratinocytes that are affected, skin cancer could also originate from the skin melanocytes. Skin melanocytes are melanin-producing cells located in the stratum basale. Melanin, the skin pigment, acts as a natural sunscreen due to its protein structure that could scatter UVB light [1]. When UV radiation triggers genetic mutations in melanocytes, uncontrolled cell division of melanocytes occurs [9,18]. Thus, malignant melanoma (MM) is the malignant neoplasm that originated from melanocytes and is highly metastatic [1,9]. It is the most aggressive and lethal form of skin cancer, with a rising trend of new occurrence in the regions of light-skinned populations with excessive sunlight exposure [10,19]. Worldwide, skin melanoma is the 18^th^ most prevalent cancer among the 35 types of cancer with nearly 325,000 new incidences in 2020 [20]. It contributes 60% of mortality as a result of skin cancer [3]. The main risk factor of MM is UV light exposure. In addition, intermittent intense sunlight exposure is related to a higher risk of melanoma than a lower level of chronic sunlight exposure [4,21]. Other risk factors include skin type (light skin), the number of atypical, naive, older age, family history in first-degree relatives, and gender (male is highly susceptible than female). Dermoscopy, a non-invasive method, is most effective in diagnosing and early detecting NMSC and melanoma [19,22]. The mnemonic ABCDE rule of melanoma (asymmetry, border, color, dimension, and evolution) has been utilized to identify melanoma [9,19]. Ugly-duckling signs would be helpful in the case of patients with multiple nevi [9]. Early detection and treatment are crucial as the treatment is more difficult when the melanoma has metastasized [18]. A study demonstrated that the countries with a very high human development index (HDI) had a higher melanoma risk than less developed countries. Thus, the implementation of public health measures has been urged to avoid a further rise in cancer incidence, especially in countries with very high HDI, including New Zealand and Australia [14].

### 1.2. History of Treatments and Limitations of Convention Dosage Form for Skin Cancer Treatment

The growing occurrence of skin cancer worldwide raises the need for multiple treatment options [10]. The well-established treatments for skin cancer include chemotherapy, immunotherapy, surgical excision, radiotherapy, Mohs micrographic surgery (MMS), and cryotherapy.

Chemotherapy involves using cytotoxic drugs to increase the life span of patients with improved quality of life (QoL) [23]. Only a small portion of drugs can reach the therapeutic site after oral administration, reducing the therapeutic efficacy. To compensate for the loss in a therapeutic effect, multiple doses are required to maintain the desired drug concentration at the therapeutic site, which might result in drug resistance due to repeated drug usage and severe toxic side effects [24,25]. In a typical process of chemotherapy, cytotoxic agents are usually administered with a combination of two or more drugs which can help to tolerate the adverse effects of other adjuvants and to achieve better therapeutic outcomes [23]. With the abundance of drugs used in patients, the risk of having drug–drug interaction is inevitable. According to Buajordet et al. [26], drug–drug interaction is estimated to cause death in 4% of patients with cancer. There were five cases of using antineoplastic agents that led to fatal adverse reactions in cancer treatment.

To further illustrate more on conventional chemotherapy, 5-Fluorouracil (5-FU) is known as one of the most widely topical chemotherapeutics used for treating cutaneous tumors and works by interfering with DNA synthesis of actively dividing cells. Moreover, 5-FU is not effective in treating invasive NMSC, superficial BCC, and SCC. The ineffectiveness in treating NMSC is due to the poor penetration into the dermis. BCC and SCC are due to the potential remaining of deeper and more invasive tumors in following treatments [27]. The currently available conventional immunotherapy used is intra-lesional interferon (IFN), where there is an initiation of apoptosis of BCC cells through CD-95 ligand–receptor interaction. Moreover, it stimulates upregulation of interleukin-2 and inhibits the production of interleukin-10, which helps in tumor regression [28]. IFN requires multiple intra-lesional injections, where the common regimen lasts for 3 weeks with 3 injections per week. The therapy is only used under specific conditions, such as patients who cannot tolerate surgical procedures [27]. Furthermore, IFN treatment can lead to side effects, such as headache, myalgia, fever, and leukopenia, limiting its usefulness in treating skin cancer [27,28]. Another example of a conventional chemotherapy is imiquimod, which is a topical immune-response modulator that is used for treating small nodular BCCs, superficial BCCs, and SCC in situ [27,28]. Imiquimod binds to cell surface receptors, such as Toll receptor 7, and stimulates the secretion of cytokines and activation of Th-1 cell-mediated immunity, resulting in increased immunostimulation [27,28]. According to Marks et al., the most common local skin reactions after applying topical imiquimod 5% cream were erythema, scabbing, erosion, and flaking. However, these side effects were well tolerated by the majority of the patients [29]. There was a total 99 patients enrolled in the 6-week study and 92 patients in the 12-week study with the requirement of applying 5% imiquimod cream or placebo cream. It was found that imiquimod is an effective agent against nodular BCC, but the tumor clearance rate of 71–76% is considered low [30].

Aside from conventional chemotherapy and immunotherapy, many other treatment options are available, such as radiotherapy, cryosurgery, excisional surgery, and MMS. Surgical methods remain as the mainstay treatment due to having a better removal rate. The most common therapy for NMSC treatment is excisional surgery, which is useful in removing low-risk tumors due to its cost-effectiveness and acceptable cure rates. This method allows histopathological examination of tissue and removal of low-risk tumors with a margin of 4 mm [27]. For cryosurgery, liquid nitrogen is used at −196.5 °C, as it destroys tumor cells through freezing and vascular stasis, and it is preferably used for treating NMSC. However, other surgical procedures are preferred over cryosurgery, due to their limit of undergoing margin analysis [27]. Moreover, surgical excision alone is curative for most patients, but the decision to start radiotherapy requires other considerations, such as tumor location, long-term function, or long-term cosmesis. Radiotherapy is usually indicated for patients with contraindication to surgery or patients having cancer in areas where the surgical procedure may lead to poor cosmetic or functional outcomes [31]. Next, MMS is one of the many treatment options for skin cancer where serial horizontal sections of the tumor cells are removed, mapped, processed, and analyzed microscopically. The surgeon examines, evaluates, and removes the tumor region until every margin of the cancer cell is cleared off [32]. This treatment provides an advantage over other excisional surgery due to its better cosmetical and functional reconstruction [33]. However, only limited surgeons are trained to carry out this procedure, and some patients cannot tolerate the procedures, even under local anesthesia [27,34]. This is due to the scarred tissue resulting from previous radiation therapy being unable to achieve adequate anesthesia, affecting patients’ pain tolerance during surgery [34]. Therefore, this procedure should only be used for patients with special indications.

As mentioned earlier, the available treatment options are inevitable for the patients to experience detrimental side effects. Patients with multiple regimens might be susceptible to drug–drug interaction or even suffer from unwanted side effects. Meanwhile, for surgical methods, patients might face poor cosmetic or functional outcomes, such as tissue scarring after undergoing a surgical procedure. Therefore, ideal drug delivery to the cancer site is preferable to achieve sufficient drug concentration at the site of action and penetrate specifically to the tumor cells to minimize unwanted systemic toxicity and have improved efficacy and safety as compared to conventional treatment options [2].

Currently, available review articles mainly focus on nanotechnology in the treatment of skin cancer. This review article emphasizes recent trends of vesicular drug delivery on skin cancer.

## 2. Methodology

Different databases were explored, such as PubMed, ScienceDirect, and Google Scholar, to search relevant articles reporting outcomes on skin cancer treatment with nanocarriers, especially with vesicular nanocarrier. Keywords used for search are skin cancer, skin malignancies, malignant melanoma, non-melanoma skin cancer, nanocarriers, liposomes, micelles, niosomes, nanoemulsion, vesicular drug delivery system, and nanogels. Full-length papers of shortlisted articles were collected to include in this review. After the collection of articles, duplicates were removed, and the articles’ title was carefully evaluated for shortlisting.

## 3. Critical Biological Barriers for Skin Cancer Therapeutics

To further understand the pharmacokinetics and pharmacodynamic of formulations, it is important to analyze the compartments of complex biological barriers, as they are one of the few primary aspects in achieving an effective therapeutic effect. In the skin, the main biological barriers that play a role in topical delivery are namely stratum corneum (SC), tight junctions (TJ) of the interfollicular epidermis, and hair follicles [35,36,37]. Moreover, another important barrier is blood vessels, which can be considered the “last barrier” of the skin [35]. With all the biological barriers found in human skin, it is vital to analyze the complex skin barrier system to understand what factors can limit the loss of water or solutes, which can help in formulation absorption to increase the therapeutic efficacy of drugs.

**Figure 1:**
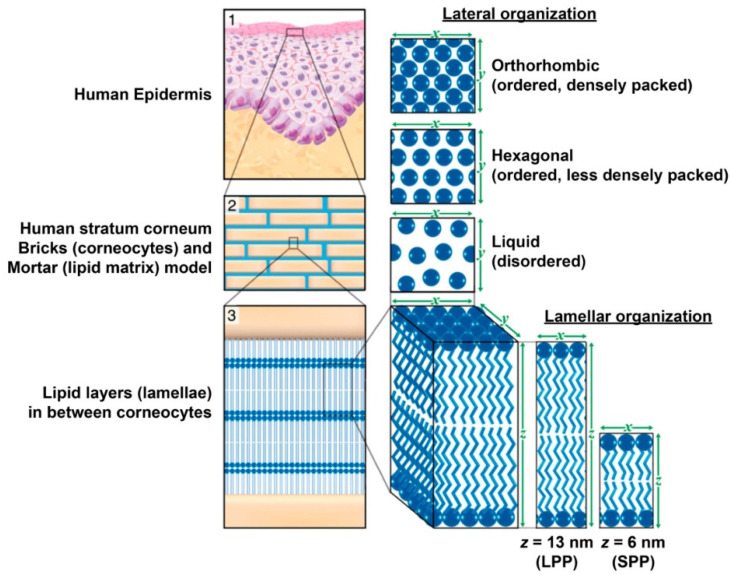
Intercellular lipids of the stratum corneum organization. LPP, long periodicity phase; SPP, short periodicity phase. Figure adopted with permission [46].

SC is the first barrier encountered by topically applied molecules and is often known as the rate-limiting barrier for topical delivery that prevents molecules from penetrating further barriers in the epidermis. SC consists of a few layers of corneocytes with cornified envelopes, intercellular lipids, and corneodesmosomes (Figure 1) [36]. Corneocytes are terminally differentiated keratinocytes characterized by the absence of cell nuclei and organelles, cytokeratin filament accumulation bundled by filaggrin, and cornified envelopes. Cornified envelopes are built of several proteins that are crosslinked by transglutaminases [36,38]. Meanwhile, in the extracellular area of SC, it is filled with lipid lamellae containing cholesterol, free fatty acids, and ceramides, which can then form two types of crystalline lamellar phases, namely the long periodicity phase (LPP) and short periodicity phase (SPP) [39,40]. Barrier function is defined by the composition of lipids, especially ceramides, such as the occurrence of lateral packing of lipid lamellae, which can be affected by skin hydration, temperature, solvents, and penetration enhancers [35]. It was found that drugs are mainly transported in the intracellular lamellar regions, where the barrier function depends on these lamellar phases [40]. Many reports have been published over time to prove the importance of SC as a skin barrier. For example, tape stripping where several layers of SC are removed has shown increased penetration of externally applied substances due to the disrupted SC with impaired skin-barrier function [41]. Besides the structural components, another aspect of SC is SC hydration, where the natural moisturizing factor plays a significant role in skin barrier function. SC hydration is responsible for the morphology and plasticity of the skin and affects the activities of proteins involved in the desquamation and synthesis of lipids [35].

**Figure 2:**
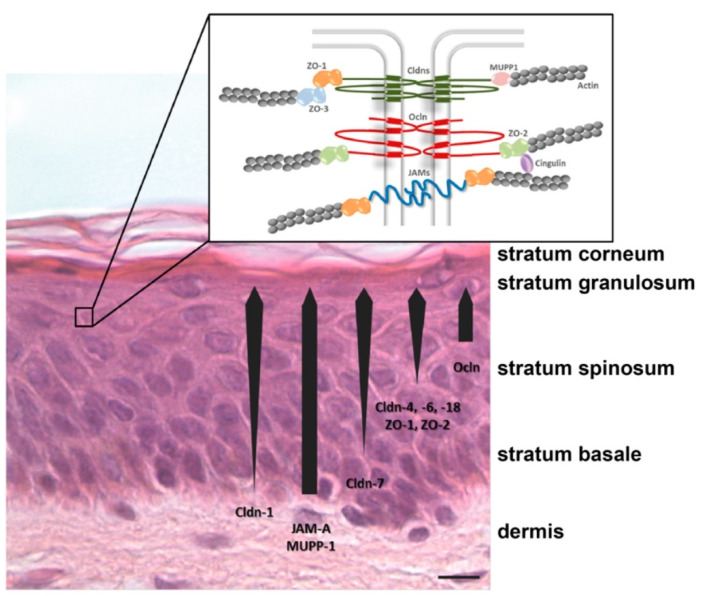
Structure of tight junction (TJ) and type of TJ proteins present in the epidermis. Cldn, claudin; JAM, junctional adhesion molecule; Ocln, occluding; ZO, zonula occludes protein, Figure adopted with permission [47].

Moreover, TJs can form the continuous barrier in the stratum granulosum of the epidermis and are known as the second barrier for the movement of molecules from outside to inside. Depending on the composition of TJs (Figure 2), they can act as barriers towards molecules of different sizes [35]. TJs consist of claudins, occluding, junctional adhesion molecule-A, angulins, tricellulin, and intracellular scaffold proteins of ZO-1, ZO-2, and ZO-3 [37]. Claudins can act as charge-selective proteins which can block the movement of ions, such as chloride ions, sodium ions, and calcium ions [42]. TJs is also a signaling platform, where scaffolding, regulation, and signaling are important parameters affected by plaque proteins, such as ZO-1, ZO-2, cingulin, and atypical protein kinase C [43]. To add on, TJs can also be found in hair follicles. Hair follicles go through three stages of hair-growth cycles, namely anagen (active growth phase), catagen (regression phase), and telogen (resting phase) [44]. Most of the hair follicles are in the anagen phase, and fewer are in the catagen and telogen phase [35]. In human anagen hair follicles, barrier-forming TJs are present from the infundibulum until the lower central part of the outer root sheath of the hair follicle [45]. The layers containing TJs in the infundibulum are covered by SC which connects to the SC of the epidermis. In addition, there is another barrier forming TJs between Henle and Huxley’s layers where these TJs are predominantly related to drug uptakes [35,45].

Lastly, the blood vessels are the last barrier for the skin, due to the endothelial cell layer responsible for the interaction between the human vascular system with the surrounding skin tissue. The role of the endothelial cell layer is to regulate permeability and vascular dilation or constriction because it can actively respond to pressure, osmolarity, heat, chemokines, and cytokine [48]. To add on, direct impact such as thermal changes can also affect the local perfusion rate. To further illustrate this, in cold stress, skin perfusion is 0.05 L/min versus 0.25 mL/min under normothermic resting conditions and more than 5 L/min in higher temperatures [35]. Therefore, the blood vessels could also serve as another critical biological barrier in the skin.

Understanding the structure and composition of skin barriers is vital in developing new drug delivery systems, where the delivery of drugs to selected skin regions is essential for treating skin carcinoma. Nonetheless, many other factors should also be considered, such as physicochemical properties, which include pharmacokinetics and pharmacodynamics, and the partition and diffusion properties of each drug intended to use. In addition, experimental biological models, mathematical, experimental models, drug preparation, drug sampling, methods of quantifying drugs, and the proper method of administration are also aspects to be considered in formulating a new drug.

## 4. Nanocarriers against Skin Cancer

Poor penetration and frequent prolonged applications are the limitations of current topical treatments [12]. Additionally, low drug aqueous solubility is the main drawback in the conventional chemotherapeutic agents. The risk of excessive toxicity due to its non-specific killing on normal healthy cells and cancerous cells is another disadvantage [49]. Consequently, patient adherence to therapy is not consistent [12]. Hence, many different types of nanoparticles (NP) are studied for skin cancer treatment to overcome these limitations associated with conventional dosage form [19]. Notably, NP demonstrates its potential in enhancing target specificity and improving permeability and retention of the drug to the tumor site. This led to lowered drug doses, minimized toxicity on healthy tissues, and enhanced patient compliance [12,49,50]. Thus, several nanocarriers, including vesicular systems, nano emulsions, nanofibers, polymeric NP, metallic NP, and lipid NP, are developed and evaluated for topical application in skin cancer treatment [12]. Various nanocarriers are discussed in this section, other than the vesicular delivery system, which is discussed in Section 4.

Nano emulsions (NEs), which show several advantages in the topical delivery of chemotherapeutic drugs in skin cancer treatment, have been extensively studied in recent years [49]. NEs are nanosized (mean droplet diameter below 300 nm) colloidal dispersions of the oil phase and aqueous-phase stabilized by surfactant molecules [12,48,51]. NE is characterized by its effective drug encapsulation, as it allows the solubilization of lipophilic drugs in the oil phase, while carrying them with hydrophilic drugs at high loading capacity [12,49]. It also allows immediate burst release, followed by sustained drug release and delivery of drugs with the desired permeation rate [49]. As NE has a small size and high surface area, it could form close occlusive contact with SC, leading to enhanced drug permeation [12,49]. The potential of NE in enhancing cutaneous localization was first investigated by Giacone et al. The NE modified with 1% *w*/*w* chitosan or sodium alginate was developed by them for topical delivery of piplartine (piperlongumine), a cytotoxic agent [50]. This study demonstrated the superiority of the nanostructured system over the simple solution in enhancing skin penetration where Dox-treated cells and DMSO-treated cells were used as a positive and negative control, respectively. The uncoated NE increased piplartine delivery to the remainder of epidermis and dermis (ED), where tumor lesion developed by 1.4-fold. NE containing chitosan (NE-C) is cationic, whereas NE containing sodium alginate (NE-A) is anionic. The interface attractive interaction between charged polysaccharides and phospholipids within nanocarriers resulted in multilayers of alternating phospholipids and hydrated polymer and/or polymer shells surrounding the oily core. By including polysaccharides sodium alginate or chitosan on NE, the skin penetration of piplartine was improved similarly (1.3–1.9-fold) despite the opposite charge. This showed that the anionic nanocarrier was similarly effective regarding skin penetration compared to cationic nanocarrier, although skin is commonly known to have a net negative charge.

Interestingly, cutaneous localization was found to be aided by the inclusion of bioadhesive polymers (chitosan/sodium alginate) to NE because the ratio of “drug in the skin (SC+ED)/receptor phase” was increased by 1.4- to 1.5-fold as compared to plain NE. Oleic acid was added to NE-C as a penetration enhancer. Inclusion of oleic acid to NE-C (oil phase containing 3:1 ratio of tricaprylin to oleic acid, NE-C-OA) showed a further increase in drug penetration (~1.9- to 2.0-fold). Moreover, NE-C-OA demonstrated a more significant skin localization by increasing absolute drug amount at the site of action by ~15-fold, although there was a concurrent rise in transdermal delivery [50]. This could be due to the lipophilic nature of piplartine that has a preferential affinity to skin and/or formulation components partitioned in tissue [50,51].

Based on the visualization of skin penetration of fluorescent compounds, the results supported NE superiority, whereby there was a 73% increase in fluorescent staining intensity when treatment with NE-C-OA than the solution and almost a 2.4-fold higher penetration depth reaching viable epidermis than solution-mediated penetration. Based on a short-term stability study, NE-A formulation demonstrated a more pronounced droplet size and lower NE electrical stabilization enhancement effectiveness than NE-C formulation. In contrast, NE-C-OA showed an acceptable stability profile among the three oil phases tested [50,51]. In 2D cancer cell cultures, piplartine cytotoxicity against melanoma cells was elevated by ~2.8-fold when NE-C-OA delivered the drug compared to similar drug concentrations in solution. The piplartine concentration required to decrease cell viability to 50% (IC_50_) by NE-C-OA was 5.1 µM, whereas IC_50_ was 14.6 µM by piplartine solution [50]. Consistent results demonstrating the cytotoxic effect were observed in a bioengineered melanoma model in which piplartine-loaded NE-C-OA caused a decrease in cell number and disorganization of the epidermis. However, the importance of targeted delivery of piplartine via topical route should be emphasized. This study also revealed that piplartine killed non-tumor cells in the 3D cancer cell model [50]. Therefore, this study supported the potential of chitosan-modified NE loaded with piplartine as a new therapeutic option in skin cancer management.

The anticancer drug 5-FU, which is applied topically in NMSC, is associated with severe side effects and very low bioavailability with oral formulation [52,53]. Current commercial topical 5-FU preparations demonstrated poor skin drug permeation and retention since 5-FU belongs to BCS class III. In this context, Ahmad et al. developed a novel carbopol based 5-FU-loaded nanoemulsion gel (5-FU–NE-Gel) and 5-FU–NE topical formulation for skin cancer chemoprevention [53]. Solubilization of 5-FU in the preparation of w/o NE is influenced by the oil phase, surfactant, and co-surfactant. In this study, surfactant (Transcutol HP with very low HLB-value), co-surfactant (PEG-400), and castor oil (oil phase) were selected for NE preparation, because 5-FU showed the highest solubility in these excipients. Moreover, 5-FU–NE3 (aqueous phase of 10.0 mg 5-FU; 20% *w*/*w*, 40% *w*/*w* castor oil, 27% *w*/*w* Transcutol HP, 13% *w*/*w* PEG-400, 20% *w*/*w* water and S-mix ratio 1:1) nanoformulation was selected as optimized-5-FU–NE because it is the most stable nanoformulation and demonstrated smallest globule size, viscosity, refractive index, and polydispersity index value with maximum droplet sizes uniformity and optimum zeta potential. Furthermore, 5-FU was mainly entrapped inside the NE core with no chemical interaction with other NE ingredients [53].

Additionally, carbopol 934 was utilized to convert 5-FU–NE3 into a gel. Based on in vitro release studies, optimized-5-FU–NE-Gel and optimized-5-FU–NE caused an initial burst release of 5-FU and subsequently a sustained release. The extended-release of 5-FU observed in 5-FU–NE-Gel is due to the formation of the matrix-type reservoir by carbopol polymer. According to the permeation study, the 5-FU permeation from 5-FU–NE-Gel was 1.56-fold greater than 5-FU–NE for the rat skin model and 12.51-fold more than the 5-FU-saturated aqueous solution (5-FU-S) for the goat and cow skin model. These findings were contributed by their smallest droplet size and viscosity and optimum concentrations of PEG-400, castor oil, and Transcutol HP. Furthermore, 5-FU–NE3-Gel showed the highest 5-FU amount localized in viable part of goat and cow skin model than other NE formulations. The formation of the intra-matrix system by carbopol gel has caused 5-FU deposition in the deep skin layer with a decline in 5-FU systemic toxicity. The in vitro therapeutic efficacy of both optimized-5-FU–NE3-Gel and optimized-5-FU–NE3 were evaluated by using melanoma cancer cell lines (SK-MEL-5-type) compared with drug solution (positive control) and blank emulsion (negative control). The cytotoxicity of optimized-5-FU–NE3 (61.75%) and optimized-5-FU–NE3-Gel (79.22%) were significantly higher than 5-FU-S, indicating both nanoformulations showed very potent therapeutic effectiveness for skin cancer treatment. The increment of cytotoxicity observed in 5-FU–NE3-Gel was due to improved cellular uptake of 5-FU by endocytosis [53]. In short, both studies have demonstrated the therapeutic potential of NE and even NE-Gel in delivering anticancer drugs (piplartine and 5-FU) via the topical route in the skin cancer treatment

The next type of nanocarrier to be discussed is polymeric micelles and NP. The current therapeutic approaches for melanoma, including photodynamic therapy, molecule targeting, radiotherapy, and chemotherapy, are expensive and unaffordable for most patients [54]. For topical chemotherapeutic delivery, nanogel seems to be a promising and cost-effective alternative for patients [55]. Recently, Sahu et al. engineered 5-FU encapsulated pH-responsive biodegradable chitosan nanogels (FCNGL) by ionic gelation methods for topical chemotherapy against melanoma [54]. Elevated drug-loading efficiency is achieved by using a chitosan–pluronic 127 nanogel system with a simple synthesis mechanism without polymerization. It is reported that the swelling capacity of FCNGL at acidic pH significantly increases because of enhanced water absorption and ionic attraction as a result of the protonation of -NHCOCH_3_ and -OH group in chitosan NP. Hence, chitosan containing amino and hydroxyl groups are crucial for pH-responsive, targeted, and topical biphasic release of 5-FU from FCNGL in an acidic melanoma microenvironment (pH 5.5–6.5). Moreover, the 55% entrapment efficiency of 5-FU to FCNGL is suitable for desired in vivo drug dosing. Meanwhile, FCNGL possesses efficient 5-FU delivery to melanoma cells due to its potent degradation capacity by lysosomal enzyme and optimum nanosized particle size distribution, reflecting compatibility for intracellular drug delivery at the melanoma site [54].

This nanoformulation also demonstrated remarkable ex vivo penetration efficiency and retention by using porcine skin, because of the interaction between cationic outer chitosan core and anionic lipids and keratin. Overall, this implicates the cationic chitosan nanogel system could potentially benefit topical delivery against skin cancer. Apart from that, noticeable side effects, such as hemolysis are observed with 5-FU topical gel application. In contrast, FCNGL showed no blood hemolysis, suggesting it as a safe medication, maximizing drug delivery to the melanoma environment, and minimizing the direct exposure of 5-FU to blood circulation. MTT assay revealed that FCNGL, blank nanogel (negative control), and plain 5-FU (positive control) demonstrated cytocompatibility at all concentrations on the human keratinocyte (HaCaT) cell line. The anticancer effect of FCNGL is evaluated on dimethyl benzene anthracene (DMBA)-induced melanoma in mice models. The topically applied low-dose FCNGL (0.2% 5-FU *w*/*v*) exhibited a significant tumor inhibition profile and a minimum mortality rate of 21% compared to the marketed high-5-FU-dosed formulation (5% 5-FU *w*/*v*) against the DMBA-induced melanoma animal tumor model. This may attribute to high accumulation in the tumor site via enhanced permeability, sustained release, and retention effect. This also allows once-daily application and reduced side effects compared to other topical 5-FU gel against skin cancer. Eventually, FCNGL treatment resulted in enhanced detoxifying levels in blood and antioxidant effect and regeneration of keratinized squamous tissue [54].

Similarly, a study by Zhang et al. on Apatinib-loaded NP was conducted to evaluate the inhibition of angiogenesis and tumor growth in melanoma model [56]. Apatinib, a selective competing antagonist to the VEGF receptors (VEGFR), specifically VEGFR-2, disrupts the ATP binding onto the VEGFR-2 expression cells. The NP was prepared by the emulsification-solvent volatilization method. From the study, the size of the nanoparticle was between 100 and 200 nm with a weakly negatively charged surface. The results showed that the NP had a controlled release rate and could sustain stable blood concentration even after initial high release. According to the in vitro study on the inhibitory effect of Apatinib NP, there was a concentration-dependent cytotoxic effect observed on the tumor B_16_ cells whereby 40 μg of Apatinib loaded into Apa/p NP showed the minimal survival rate. Meanwhile, for an in vivo study, mouse melanoma models were used, and it was found that the PLGA nanoparticle showed effective inhibition on tumor growth. The tumor weight can explain why this was lowest by using the PLGA group. The tumor volumes were smallest when using Apatinib at a concentration of 6mg compared to drug solution (positive control). To add on, tumorous tissue necrosis was visible, especially within the middle of tumor tissue where the necrotic site has a light red region, and complete cell structures were not observed. This result suggests that Apatinib-loaded in nanoparticles could inhibit tumor growth by local hypoxia and ischemia of the tumor tissue. Therefore, Apatinib-loaded PLGA/Poloxamer 407 NP showed a better inhibitory effect on tumor cells than free Apatinib, and this NP can be used for MM treatment [56].

Nanofiber has also been explored for skin cancer treatment as it provides the functional domain in flexibility design [57]. Thus, it releases a therapeutic payload at the goal site when it reacts to the surroundings. In this context, Janani et al. prepared molybdenum oxide–polycaprolactone nanofiber (MOL–PCL fibers) containing NP as a scaffold [58]. The average diameter fiber-containing nanoparticle was larger than control PCL fiber due to the establishment of nanoparticles inside the nanofibrous scaffold. Moreover, MOL–PCL fibers are types of application that conductivity needed lesser to induce the conductivity of the natural polymer and the process of electrospinning is suitable to make them. The designed scaffold reduced the cell viability of skin cancer by more than 50%, using Acridine Orange (AO)/Propidium Iodide (PI) staining and the probable mechanism via apoptosis. Furthermore, the cancer progression of zebrafish was found and reduced by more than 30% in 14 days in vivo study [58].

On the other hand, Suneet et al. prepared a magnetic Fe_3_O_4_ nanofibers-based bandage [59]. On the external application, the heat energy locally was dissipated by the polycaprolactone-Fe_3_O_4_ fibrous mat-based bandages. Moreover, it changed the magnetic field and enhanced the temperature of the environment in a controlled way. In vivo study confirmed that increased temperature could kill doxorubicin (Dox)-resistant and parental Hela cells. It is because, as the temperature increased, the Dox’s activity increased. Therefore, it was caused parental Hela cells dead (≥85%) [59].

Gold nanoparticles (AuNPs) are metallic nanoparticles that have been investigated as potential drug carriers for topical delivery of the anticancer agent. The overall benefits of AuNPs include the ease of synthesis, conjugation of appropriate biomolecules on the surface of nanoparticles with no alternation to the biological properties, and good biocompatibility; they also do not elicit significant immune responses [60]. These properties were explored for theragnostic applications for skin cancer. The conjugation of appropriate biomolecules and drugs on the AuNPs allows for the tumor localization at the early stage. It enables detection and targeted drug delivery towards the tumor cells. A study was performed to investigate the effect of layer-by-layer polymer-coated gold nanoparticles (AuNPs) containing imatinib mesylate (IM) for the treatment of melanoma. The developed AuNPs had an average particle size of 98.5 nm, and the loading efficiency was around 28.3%, which is the greatest loading capacity seen in AuNPs. A skin penetration study was conducted by using porcine ear skin, as the in vitro model showed a 6.2-fold enhancement of the skin penetration in IM-loaded AuNP as compared to free IM (positive control). The tape-stripping study of the human SC also showed the IM-loaded AuNP retained 7.8-fold more IM in the SC than the free drug after subjecting to iontophoresis. Moreover, IM-loaded AuNP significantly reduced the cell viability of B16F10 compared to free IM with *p*-value < 0.001 [61].

Similarly, superparamagnetic iron oxide nanoparticles (SPIONs) have been investigated as a potential vector for transdermal drug delivery of epirubicin (EPI) to treat skin cancer. The drug-release profile showed that the drug release in EPI–SPIONs is pH-responsive. The greatest release was observed at a pH 4.8, similar to the pH of tumor tissue. In contrast, the amount of EPI released from the nanoparticles at a normal physiological pH level (7.4) was minimal. This indicates that the EPI–SPIONs are more selective towards the tumor and readily release in a more acidic environment. The cytotoxicity study of EPI–SPIONs involved in WM266 (melanoma cells) demonstrated an inhibitory effect on melanoma cell proliferation, and it appeared to be dose-dependent.

Moreover, the study also showed the EPI–SPIONs are biocompatible with human keratinocyte HaCaT cells. The iron oxide exhibits magnetic responsiveness and has a role in skin permeation enhancement by using magnetism. The in vitro transdermal studies with human abdominal cadaver skin demonstrated that the EPI–SPION enhanced the penetration deep into subcutaneous tissue via follicular pathways after being subjected to an external magnetic force. The enhanced permeation and targeted drug delivery of SPION made it a feasible carrier for the treatment of skin cancer [62].

During the early 20th century, solid lipid nanoparticles (SLNs) were suggested as a possible alternative to liposome, emulsion, and polymer nanoparticle delivery methods. They are versatile nanocarriers that can improve the therapeutic effect of various molecules, due to their low toxicity, high bioavailability of drugs, high stability, versatility when incorporating drugs, and feasibility of large-scale production [63]. In this context, researchers have embedded 5-FU into a highly penetrating shell-enriched nanoparticle to achieve drug targeting on tumor cells. Results showed that 5-FU-loaded SLN increased the penetration capability of 5-FU through a modified Franz cell system and, hence, increased the drug effectiveness. The marked effectiveness was observed in histopathological studies. In the treatment group of male BALB/c mice, there was a significant reduction in inflammatory reaction and small hemorrhagic areas compared with controls and free 5-FU groups [64]. In another study by Geetha et al., they tested the use of the antioxidant sesamol in SLNs to treat skin cancer, because sesamol is proven to have an excellent reactive oxygen species (ROS) scavenging capacity [65]. Cream-based topical preparations were formulated to incorporate free sesamol with SLNs. The in vitro studies were performed on Molt-4 and HL-60 cancer cells lines to determine anticancer abilities. It has been found that DNA fragmentation, a marker for apoptotic cell death, can be observed in HL-60 cell lines but not in Molt-4 cell lines. In vivo studies on LACA mice with skin, carcinogenesis was performed to determine the antioxidant activity and to carry out anticancer studies, permeation studies, and skin retention studies. The studies have shown that SLNs provided higher drug retention in the skin and increased effectiveness in delaying the onset of skin tumors compared to free sesamol (positive control). They also showed higher stability, desirable spreadability, and prolonged duration of action compared to formulations of SLNs in bases other than cream-based topical treatment [65].

In short, various studies have demonstrated the therapeutic benefits of different types of nanocarriers in skin cancer treatment via the topical route of administration. The summary of the recent studies of nanocarriers for skin cancer therapy, as discussed earlier, is listed in Table 1.

## 5. Vesicular Drug Delivery for Skin Cancer Treatment

**Figure 3:**
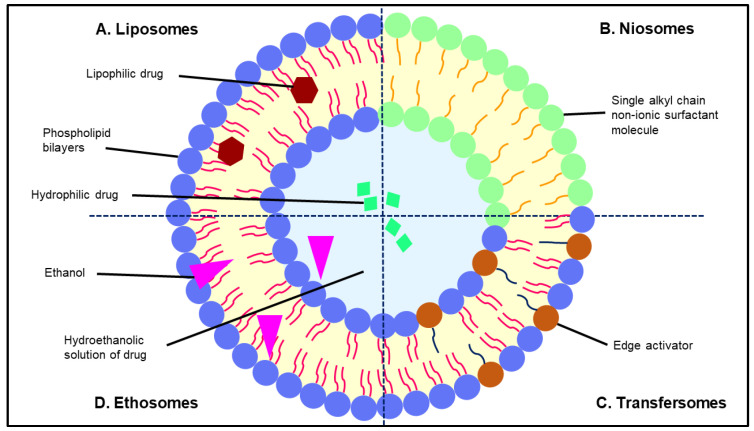
Schematic representation of vesicular drug delivery systems, namely (**A**) liposomes, (**B**) niosomes, (**C**) transfersomes, and (**D**) ethosomes.

Topical drug delivery offers an effective and convenient dosage form for the treatment of local pathological conditions, including skin cancer [66]. In recent years, the vesicular drug delivery system has gained much attraction to be developed as a novel topical drug delivery platform for the treatment of skin cancer. Vesicular drug delivery is developed when the amphiphilic building blocks self-assemble in the presence of water, leading to the formation of highly ordered assemblies with at least one concentric bilayer [67]. This section discusses the therapeutic potential of the vesicular drug delivery systems, namely liposomes, niosomes, transfersomes, and ethosomes (Figure 3), in skin cancer treatment.

### 5.1. Liposomes

Liposomes are phospholipids with a dimension of 50 to 100 nm and have an internal aqueous process similar to the arrangement of the membrane of biological membranes. Hence, they are excellent delivery systems as they resemble the phospholipid bilayers of body cells. An important improvement in the pharmaceutical industry is the prospect of connecting the liposome surface with ligands and or polymers [68]. In terms of cancer treatment, liposomes are known to linger near the tumor interstitial fluid [69]. Many liposomal preparations containing anticancer medications have been formulated, including treatment for melanoma. Several agents can use the surface functionality of liposomes to resolve biological and physiological obstacles to nano-carriers. For example, polyethylene glycols (PEGs), antibodies, aptamers, proteins, peptides, and ligands can be attached to the liposome surface to functionalize the liposomes; moreover, increasing the circulation time ligands can achieve targeting to specific cells [70,71]. Both selective drug delivery and controlled release have been accomplished with success in liposomal vesicle growth. This property is useful when treating cancers, such as skin cancer, as the first-line treatment of cancer is chemotherapy, among other methods, such as surgical resection and radiation therapy [72].

Chemotherapy is the main treatment for melanoma, an aggressive and most severe type of skin cancer. However, it comes with strong side effects, due to its cytotoxicity, which also affects non-cancerous cells, and its poor targeting of malignant cells. Drug resistance is also an issue, especially with single chemotherapeutic drug treatment. For example, pro-apoptosis agent tumor necrosis factor-related apoptosis-inducing ligand (TRAIL) can induce drug resistance, due to its low death receptor expression levels [73]. Studies have shown that combination therapy of chemotherapy agents could reduce drug resistance and increase therapy efficiency [73]. A study by Huang et al. used a combination of paclitaxel (PTX) and TRAIL with a liposomal drug delivery system to achieve an increase in anti-melanoma effect, targeted drug delivery (hence, reducing toxicity), and tumor microenvironment responsiveness [73]. TRAIL attaches to the negatively charged liposome surface, and PTX is encapsulated within the liposome. The liposomal complex also contained a stearoyl chain (C18) fused with cell-penetrating peptide (TR) that is sensitive to pH. TR can achieve melanoma cell targeting as it binds specifically to integrin receptors ɑVβ3 which is rich in melanoma cells. It can then release TRAIL in a low pH microenvironment, as the liposomal charge is reversed in acidic conditions and facilitate the internalization of liposomes into cells.

Although recent studies show that melanoma is highly vulnerable to TRAIL-based drugs because of its low expression levels of death receptors, the combination of TRAIL with other chemotherapeutics, such as PTX, has been proven to achieve high synergistic effect against tumors. In this study, mice were used as subjects, and the rate at which the drug was loaded was calculated and then measured by HPLC to ensure that the amount of drug was above the therapeutic level for the subjects’ body mass. The liposomes co-loaded with PTX and TRAIL showed an improved stability and drug release profile. The delivery of TRAIL and PTX to tumor cells were selective and pH-sensitive, and it also showed significant improvement in drug biodistribution in B16F10 bearing C57 BL/6 mice. Results also showed higher anticancer efficiency when assessed with the MTT assay against B16F10 cells. The formulation was robustly safe in terms of safety, as the body weight and blood cell count of mice in all groups remained unchanged [73]. Hence, liposomal TRAIL–PTX has shown significant anticancer efficiency when tested on the animal model without any adverse effects.

Other than a combination of chemotherapy drugs, systemic chemotherapeutic agents are also currently being implemented with immunotherapy for the effective treatment of skin cancer. Moreover, 5-FU is a potent chemotherapeutic medication that can be administered systemically or topically against skin cancer and pre-cancer lesions [74]. In this regard, 5-FU was paired therapeutically with cetuximab, an immunoglobulin G (IgG) antibody with an inhibitory effect on EGFR affinity. Cetuximab is essentially an IgG1 monoclonal antibody. In the treatment of cancer, it inhibits EGFR through a high-specific bond, which leads to the inhibition of EGFR tyrosine kinase activity and, hence, the interruption of the cell cycle. Petrilli et al. formulated a topical treatment of cetuximab functionalized liposomal complex encapsulating 5-FU for skin cancer [74]. The new formulation was immunoliposomes encapsulating 5-FU conjugated with cetuximab. It was compared to controls without cetuximab (negative control) and liposomes with 5-FU without conjugation with cetuximab. In the SCC xenograft model, the therapeutic effectiveness of the administration route using the thematically applied iontophoresis and subcutaneous injections of 5-FU immunoliposomes were tested [74]. In vitro studies on cellular uptake were performed on A431 (EGFR positive) and B16F10 (EGFR negative) cell lines. EGFR positive cell lines showed higher cellular uptake of therapeutic agents than EGFR negative cell lines. In EGFR positive cell lines, there was a 3.5 times increase in absorption of cetuximab immunoliposomes cellular uptake than control liposome. Skin penetration tests have shown that immunoliposomal iontophoresis doubled 5-FU penetration of the viable epidermis as opposed to the same control liposome therapy. The formulation was administered in mice via iontophoresis and subcutaneous injections and the volume of SCC tumors was observed after 22 days of treatment. Subcutaneous immunoliposomes injection decreased the volume of the tumor by more than 60% relative to the negative tumor and about 50% compared to 5-FU and liposome-treating systems in vivo [74].

**Figure 4:**
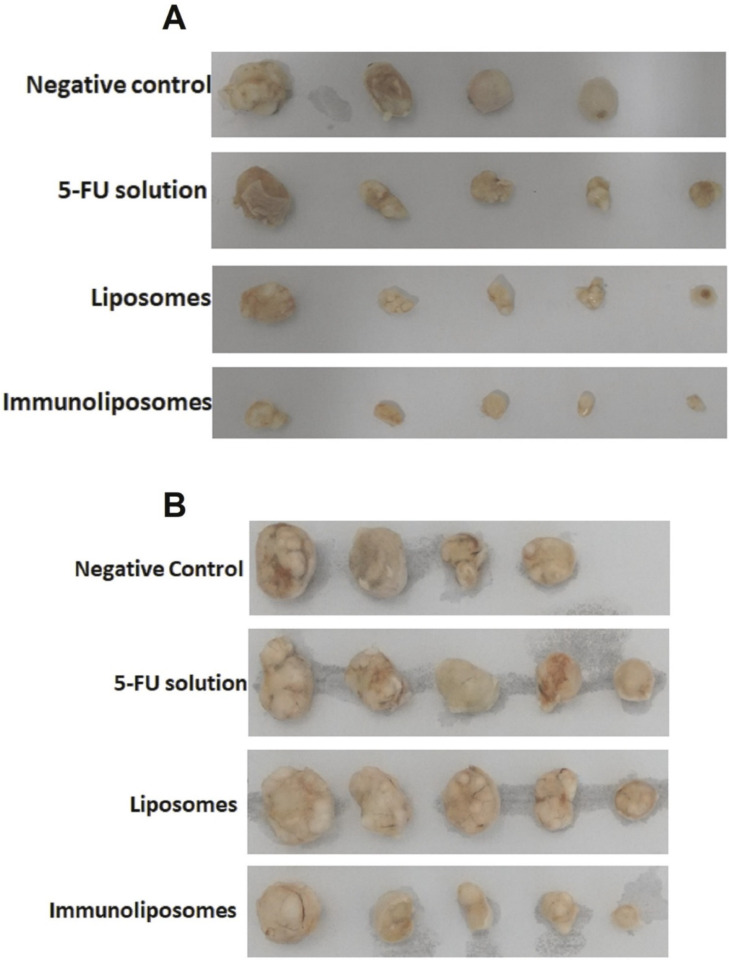
Tumor sizes after treatment by (**A**) iontophoresis or (**B**) subcutaneous injection. Replication in each treatment group was placed in a line [74].

Interestingly, topical iontophoresis administration has more significant tumor reduction by approximately 2-fold relative to control liposome and 5-FU solution subcutaneously. It is also highly efficacious for immunoliposome therapy. The tumor reduction for different groups can be seen in Figure 4. However, histological research found that an immunoliposome iontophoresis is more successful in inhibiting cell proliferation than the subcutaneous injection, which results in cells that have less violent features [74]. This study showed that cetuximab–immunoliposomes can significantly increase the cellular uptake into EGFR-positive skin cancer cells. The iontophoresis process for both liposomes and immunoliposomes was also beneficial in reducing permeation of 5-FU through the skin, hence reducing unwanted systemic effects. Overall, immunoliposomes delivered by iontophoresis were proven to be the most beneficial in skin cancer treatment, as they can control tumor growth and reduce cancer cell proliferation in skin cancer.

Next, vemurafenib (Vem) is a recently FDA-approved chemotherapeutic agent for the treatment of melanoma. It works by inhibiting mutated genes in melanoma, especially the BRAF V600E gene [75]. However, it is usually delivered orally, causing various unwanted adverse effects to the body, such as damage to the liver and kidney. In this context, Zou et al. prepared a topical formulation of Vem encapsulated in a liposome that has been peptide-modified (Vem–TD–Lip) to achieve specific inhibition of subcutaneous melanoma, while preventing the occurrence of unwanted systemic effects [75]. TD (ACSSSPSKHCG), a biologically inspired peptide, was used as a permeation enhancer, as it can temporarily open up the paracellular pathway to allow for drug delivery across the skin layers. The size of Vem–TD–Lip was found to be 105.66 nm on average and had a loading efficiency of 98.92% [75]. The results showed that liposomes’ formulation could be internalized to A375 cells instead of control groups due to the difference in zeta potentials. Selective inhibition of Vem was studied on three different cell lines: human melanoma cells (A375), murine melanoma cell line (B16F10), and human umbilical vein endothelial cells (HUVECs). Results showed that only A375 cells had decreased viability, whereas the other two cell lines showed no cytotoxicity. This shows that Vem only inhibits human melanoma cells. Inhibition ability was also shown to increase when the liposomal drug delivery system was used with Vem. Permeation studies indicated that the permeation ability across skin increased significantly when TD is present in liposomes encapsulating Vem, as compared to drug solution (positive control). This shows that the presence of TD affects the penetrating ability across the skin. TD also affected the number of drugs delivered to cells. This can be seen when the number of drugs delivered was significantly higher in Vem–TD–Lip compared to the Vem–Lip group. When the formulation was given orally or by intravenous injection, there were significant adverse effects on the liver, kidney, and lungs. However, when applied topically on the skin, the formulation was safe and showed no significant adverse effects [75]. It also displayed desired antitumor ability as there was a decrease in concentration of encapsulated tumors treated with Vem–TD–Lip group compared to the control groups. The study has provided a new and effective mechanism to deliver Vem that can achieve drug targeting and inhibit subcutaneous melanoma.

Apart from chemotherapeutic agents, siRNA can also be potentially beneficial for skin cancer treatment. However, the permeability of siRNA through the SC is the major setback for topical application. As discussed earlier, most skin cancers develop due to the mutation of the BRAF protein [76]. Hence, in a study by Dorrani et al. [76], cationic liposomal formulations complexed with BRAF–siRNA were formulated to achieve permeation through the SC and deposition in the skin. Liposomal formulations were developed with different ratios of edge-activator sodium cholate (NaChol) and cationic lipid 1,2-dioleoyl-3-trimethylammonium-propane chloride (DOTAP). Skin-permeation studies were performed on dermatomed freshly removed human cadaver skin from a donor. The evaluation of intracellular localization and BRAF expression knockdown was performed on UACC-903 human melanoma cells. UACC-903 melanoma cells were used as they contain high concentrations of mutated BRAF V600E gene. Liposomes with an 8:1 ratio of DOTAP:NaChol and complexed with siRNA at 16:1 ratio showed the highest rate of skin permeation, with the majority deposited at upper dermis due to ideal characteristics and the ratio of contents in the formulation. All lipoplexes successfully internalized into melanoma cells due to the positive surface charges [76]. To measure BRAF protein expression levels in melanoma cells, an in-cell immunofluorescence assay was used. The assay revealed that they were significantly reduced when treated with the novel drug compared to control groups. Hence, it can be said that passive delivery of an edge-activated liposome delivery system can encapsulate siRNA, deliver it across SC, and deposit it at a specific site for treatment of melanoma.

In conclusion, liposomes can overcome the side effects of chemotherapy in skin cancer treatment. The use of colloidal delivery mechanisms allows for high levels of the drug to be delivered to target cells. In skin cancer treatment, liposomes are an excellent drug carrier, as they can increase the therapeutic effect of medications through drug targeting, while reducing unwanted systemic effects.

### 5.2. Niosomes

Niosomes are colloidal bio-carriers made of non-ionic surfactant-based vesicles via self-assembly [77,78]. The structural organization of niosomes’ vesicles closely resembled liposomes’ spherical bilayer structure. However, the formation of niosomes is derived from the mixture of cholesterol, single-alkyl-chain non-ionic surfactant, and charge-inducing molecules upon hydration. In contrast, the composition of liposomes is phospholipid-based [78]. The outer shell of niosomes is formed by the hydrophobic region of the surfactant, whilst the hydrophilic region of surfactant forms the core of the vesicles [77].

The topical treatment 5-FU is commonly used for actinic keratosis and superficial BCC and SCC [27]. Nonetheless, the hydrophilic nature of 5-FU has hindered the drug permeation across the skin barrier. To enhance 5-FU permeation into the skin, niosome has been proposed as the new carrier system for a more effective topical delivery. In the study conducted by Paolino et al., the mean particle size of unloaded niosomes was ~500 nm, whereas the 5-FU loaded niosomes’ size was ~200 nm. The small particle size of the carrier enabled the penetration of API into the skin barrier more easily and allowed for the delivery to the target site [79]. In addition, the drug was released in a sustained release pattern without initial burst release. It also has a high loading capacity due to the bilayer structure of niosome and its ability for drug entrapment within the vesicles. In the in vitro, human skin percutaneous study of 5-FU-loaded niosomes demonstrated an eight-times greater permeation than the aqueous topical 5-FU solution. As for the in vitro evaluation of anticancer activity, the cell lines involved are SKMEL-26 (human melanoma cell line) and HaCaT (human keratinocytes). In the experiment with SKMEL-26 cell line, 5-FU niosomes showed greater anticancer activity than free 5-FU. A maximal reduction of cell viability of 80% was observed at 10 μM niosomal 5-FU formulation, whereas, with the same concentration, the free drug (positive control) can only achieve 50% of cell viability reduction. On the other side, treating HaCaT cell lines with encapsulated 5-FU at 10 μM can result in 60% cell viability reduction [80]. This showed that the cytotoxicity of 5-FU loaded niosomes is not selective against melanoma cells; however, it induces more significant cytotoxicity towards melanoma cells than normal skin cells. In all, the niosome nanoparticle formulation of 5-FU produced smaller vesicles with greater anticancer activity as well as enhanced percutaneous permeation.

**Figure 5:**
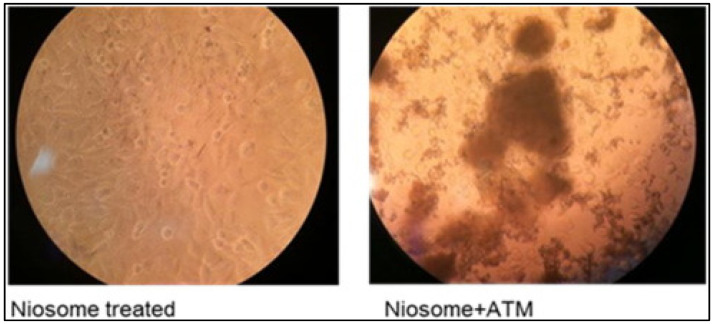
Light microscopic image of niosome-treated and ATM–niosome treated A-375 cells [82].

Another drug investigated for its anticancer effect using niosomal drug delivery system is the artemisinin derivative, artemisone (ATM). The pharmacokinetics study of the free drug demonstrated low water solubility, lipophilicity, bioavailability, and short half-life, limiting its clinical use [81]. To overcome these limitations, ATM is formulated with niosomes and was investigated by Dwivedi et al. for its efficacy against human melanoma cells [82]. The ATM-loaded niosomes that were prepared by the sonication method had an encapsulation efficiency of 67% and an average particle size around 211 nm. The result of the in vitro drug release study showed that the entrapped ATM has a prolonged release time (9 h) compared to the free drug (5 h). In addition, the slow and steady drug-release pattern in ATM-loaded niosomes suggested a homogenous dispersion of drugs within the vesicles [83]. Cytotoxicity study was performed on A-375 (human malignant melanoma cells) and HaCaT (human immortalized keratinocytes) cell lines. It showed that the ATM-loaded niosome significantly suppressed on the A-357 cells in a dose-dependent manner. The free drug inhibits (positive control) 50% of the melanoma cells’ growth at 0.06 mg/mL concentration. With the same concentration, the encapsulated ATM has a near-complete suppression of the growth of melanoma cells. In the experiment with HaCaT cells, the cytotoxic effect was minimal, and the free drug exerted greater cytotoxicity in HaCaT cells when compared to the niosome-loaded ATM. This suggests that the cytotoxicity of ATM on normal keratinocytes can be ameliorated by the niosome vesicles, as the encapsulation of ATM provided a protective effect that limits the interaction of ATM with normal keratinocytes. Moreover, apoptotic cell determination was performed on empty niosomes and ATM-loaded niosomes. The unloaded niosomes showed negligible cytotoxicity, and significant apoptotic cell death was observed in ATM-loaded niosomes. This indicates that cytotoxicity was induced by ATM, and the vesicles is non-cytotoxic (Figure 5). Overall, the sustained-release profile niosomal ATM formulation improves the drug release, limits the toxicity, and also may have selective cytotoxicity against melanoma cells [82].

In conclusion, the research results that have been discussed earlier showed the potential of niosomes’ application as the novel drug delivery system for anti-melanoma therapy. Niosomes enabled the sustained release of the drugs and also may improve the targeting of the anticancer agent. The small particles size and enhanced skin permeation also allow the administration of drug via topical route.

### 5.3. Transfersomes

Gregor Ceve first introduced transfersomes in 1991 [84]. Transfersomes consist of an aqueous core surrounded by a lipid bilayer that provides the ultra-deformable vesicle, allowing transfersomes to have extremely adaptable aggregation, self-optimization, and self-regulating properties [84,85]. Another important compartment of transfersome is the edge activator, which acts as a surfactant and allows for the destabilization of transfersome for better deformability of the transfersome membrane [86]. Various studies were performed on transfersomes with different formulations. Firstly, Khan et al. [87] studied 5-FU that was prepared in transfersome gel with different formulations to investigate improving skin absorption of 5-FU for treatment of NMSC and actinic keratosis. The conventional rotary evaporation sonication method was used in the preparation of transfersomes containing 5-FU, and Span-80 and Tween-80 as edge activators (EAs) with a phosphatidyl choline (PC):edge activator ratio (PC:EA) of 95–80:5–20% *w*/*w*. The ratio showing the highest entrapment efficiency was PC:EA of 95:05, using Tween-80 as EA in which the entrapment efficiency was 76.3 ± 1.13%. From the in vitro permeation study, the formulation TT-2 (PC:EA, 90:10) has the highest percentage of drug deposition at 81.3%, while formulation TT-3 (PC:EA, 85:15) has the highest permeation at 30.36 µg/cm^2^/h [87]. This can be explained by the presence of higher concentration of edge activators in formulation TT-3 because edge activators allow for better permeation and act as surfactants [88]. As for the in vivo study, TT-2 formulation was selected for producing transfersomal gel with 1% carbopol due to better drug deposition and better entrapment efficiency. The in vivo study reveals that newly formulated transfersomal gel had minimal hyperkeratosis than marketed formulation (positive control). The histopathological study indicates better skin tumor treatment due to better entrapment efficiency and permeation of the chosen formulation. Overall, it was found that Tween-80 acts as a better edge activator than Span-80 in skin cancer treatment, based on its entrapment efficacy and drug deposition [87].

Next, Chen et al. [89] studied the potential of carvedilol-loaded transfersomes in skin cancer prevention. The mechanisms involved in cancer prevention inhibit UV-induced oxidative stress, inflammation, oncogenic signaling pathways, and DNA damage. Thin-film hydration method was used to prepare the transfersomes at different ratios where Tween-80 and sodium cholate were used as surfactant and lipids used were L-α-phosphatidylcholine (SPC), hydrogenated L-α-phosphatidylcholine (HEPC), and 1,2-distearoyl-sn-glycero-3-phosphocholine (DSPC) [89]. As stated in previous studies, the nanovesicle’s size should be 300 nm or below to allow drugs to be delivered into deeper layers of the skin [90]. Therefore, the smallest particle size of the formulation was chosen to be further analyzed. The optimized formulation was F18 with ratio carvedilol:SPC:Tween-80 of 1:3:0.5 and was found to have a particle size of 197.11 ± 4.68 nm, PDI of 0.3 ± 0.01, the zeta potential of 15.7 ± 0.7 mV, and encapsulation efficacy of 69.7%. From in vitro drug release kinetic, it was found that, from F18, only 51% of the drug was released after 24 h. Moreover, an ex vivo skin permeation study revealed that the drug permeation of F18 was lower (14.3 ± 6.71%) than a drug-free as positive control (22.3 ± 9.11%) [89]. Even though both drug release and drug permeation were low, these traits may be advantageous if the formulation is required for local effects and a controlled release rate that can reduce systemic absorption. In addition, a mouse epidermal cell line was used for cytotoxic study and detection of intracellular uptake of the transfersome. The study proved that there was an internalization of transfersome into the cells and concentration higher than 10 µM may cause cytotoxicity. Lastly, by using UV-induced DNA damage, inflammation markers, and apoptosis on 3D reconstitution human skin model, it was found that the number of apoptotic cells was reduced in a treatment using 4 µg of F18 compared to the untreated group. This study proves that topical F18 transfersome has photoprotective effects against UV-induced DNA damage, inflammation, and apoptosis [89].

Moreover, Raahulan et al. studied the treatment of melanoma skin cancer by using PTX-loaded transfersomes [91]. A conventional rotary vacuum evaporator was used to prepare the transfersomes at different ratios in which the ratio of PC:Span-80 is in the range of 95–80:05–20% *w*/*w*. In the in vitro study, the dialysis bag method was used where transfersomal solution was suspended in a buffer solution with pH 7.4 under a constant temperature of 37±1 °C and constant magnetic stirring. It was found that the ratio of PC:Span-80 of 90:10 showed the highest percentage of drug released at 72%. To add on, this formulation with a ratio of 90:10 showed the highest entrapment efficacy at 68.2% among other formulations. This is due to the ability of Span-80 to entrap the drug in a small multilamellar vesicle, allowing high entrapment efficacy. Lastly, transfersomal gel was prepared by using carbopol 940 mixed with transfersome solution, and the resulting gel formed had a pH of 6.8, which could avoid the risk of irritation when applying the gel on skin surface [91]. From this study, it was proven that transfersome can entrap PTX at a high level for the treatment of melanoma skin cancer.

**Figure 6:**
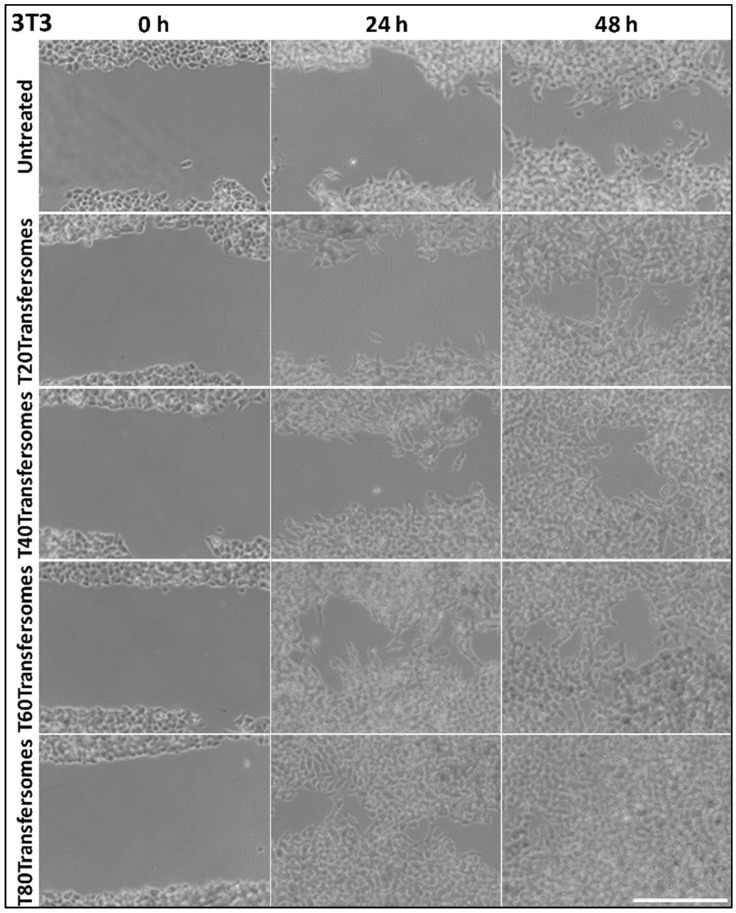
Optical microscopy images of wound closure in fibroblasts (3T3) comparing untreated control cells with cells receiving treatment with transfersomes for 0, 24, and 48 h. Bar corresponds to 500 μm [93].

In addition, vitamin E was found to be able to protect against UV-induced photodamage, as it has antioxidant and UV absorptive properties [92]. In conjunction with that, Caddeo et al. studied tocopherol-loaded transfersome to evaluate antioxidant and skin-regenerative properties [93]. Tocopherol is chosen for further investigation because alpha-tocopherol is the most commonly found isoform of vitamin E and shows a strong antioxidant property. The transfersome formulation consists of soy PC, alpha-tocopherol acetate, and an edge activator of either Tween-20, -40, -60, or -80. The mean diameter of the formulation using Tween-80 was the smallest among other formulations. To add on, the formulation using Tween-80 (HLB 15) showed the highest entrapment efficiency at 90 ± 2%, while the formulation using Tween-20 (HLB 16.7) showed the lowest entrapment efficiency at 72 ± 8%. This is because the entrapment of lipophilic drugs can be enhanced by using surfactants with a lower HLB value (2.0). As for in vitro skin-permeation study, the dorsal skin of one-day-old pigs was used and put in between donor and receptor compartments of Franz vertical cells [93]. Formulation using Tween-80 showed the highest accumulation in the skin among other formulations. Moving on to in vitro biocompatibility and antioxidant activity, epidermal keratinocytes (HaCaT) and dermal fibroblasts (3T3) were used, and it was found that the type of Tween used had no impact on biocompatibility for each formulation. As for antioxidant activity, both cell lines treated with tocopherol-loaded transfersome showed a protective effect against hydrogen peroxide. The formulation using Tween-40 showed the highest in 3T3 cells, while the treatment in HaCaT cells showed about the same result among all formulations. Next, based on in vitro wound closure study, the formulation using Tween-80 showed faster regeneration and complete wound closure in HaCaT cells and 3T3 cells (Figure 6) [93]. Therefore, it can be said that alpha-tocopherol loaded into transfersome was able to help enhance delivery into skin and Tween-80 is the best choice among Tween-20, -40, and -60, as it can produce the smallest-molecular-size transfersome and high entrapment efficiency for the delivery of tocopherol.

In the next study, Kassab et al. investigated topical photodynamic therapy using transfersomal aluminum phthalocyanine tetrasulfonate (AlPcS4) [94]. Thin-film hydration method was used to prepare transfersome containing PC with different amounts of sodium deoxycholate (SDC) where the formulations prepared were with PC/SDC ratio of 5:1, 10:1, and 15:1. For choosing the formulation used in in vitro and in vivo study, transfersome with a ratio of 10:1 was found to be the optimum formulation due to its moderate particle size and moderate encapsulation efficiency as compared to the other formulation with very poor encapsulation efficiency or too large particle size. For the in vitro photocytotoxicity study, the AlPcS4-loaded transfersome had higher phototoxicity than free AlPcS4 when tested on baby hamster kidney (BHK)-21 fibroblasts cell line. Moreover, from the histological examination of AlPcS4-treated skin, transfersome-AlPcS4 is shown to cause photodamage in deeper regions. This proves that transfersome-AlPcS4 has enhanced penetration and deeper uptake in the skin. In addition, transfersome-AlPcS4 also showed higher accumulation at the skin when compared to free-AlPcS4 (positive control) [94]. This is due to the partition of transfersome particles into the lipophilic cell membrane compartments, increasing its uptake [95]. Therefore, the AlPcS4-loaded transfersome allows better uptake into the skin for a more effective photodynamic therapy.

In short, these studies have shown that transfersome is a novel drug delivery in treating skin cancer due to the presence of edge activators that allow better penetration into the delivery site. However, it is important to note that the ratio of lipid to edge activators must be optimum to have an optimum size of the final transfersome formulation. In addition, the entrapment efficacy, which plays a major role in the delivery of selected drugs into the human body, is another important aspect in formulating transfersome.

### 5.4. Ethosomes

Ethosomes are phospholipid-based elastic NP. Phospholipids, water, and a high ethanol concentration (20–45%) are the main components in the ethosomal system. Ethanol is known as a rich permeation enhancer that increases the penetration to the skin [96]. Hence, ethosomes are suitable as drug nanocarriers in skin cancer treatment due to their enhanced skin permeability. The ethanol composition may be easier to penetrate the skin to a deeper region as it provides the vesicles with soft elastic characteristics. The presence of ethanol in phospholipid vesicles may enhance the drug penetration because it affects the double-layer structure of SC [66]. Ethanol in ethosomes could also improve the lipidic membrane fluidity of skin [97,98]. Moreover, the unique feature of ethosomes is to protect the drug from the external environment, transport the drug across the skin layers, and provide sustained drug release [97]. Thus, ethosomes can improve drug delivery for skin cancer treatment.

PTX, an anticancer drug has poor solubility and low gastrointestinal absorption. Elham et al. synthesized the PEGylated ethosomes and PTX loaded in ethosomes to improve solubility through the skin [98]. The SK-MEL-3 cell line was assessed in these ethosomes that were prepared by reverse-phase evaporation technology through their cytotoxic effect. The sonication can cause the NP to become smaller in size. However, the PEGylated ethosomal particle loaded with PTX (138.1 ± 2.7 nm) had a larger particle size when compared to the blank PEGylated ethosomal particle (102.3 ± 2.1 nm). Zeta potential became positive (13.1 mV) due to the positive charge of PTX, whereas the zeta potential of the blank PEGylated ethosomes was negative (−19.2 mV). Moreover, it showed that prolonged drug delivery to the tumor in this study due to the drug loading (2.82 ± 0.27%) and encapsulation efficiency (96 ± 1.27%) were displayed respectively. The maximum cytotoxicity in the study by PEGylated ethosomal particles and blank PEGylated ethosomes (negative control) were 1.8 ± 0.1% and 8.2 ± 1.01%, after 24 h on SK-MEL-3 cells. It also showed that 4.5-fold increased the cytotoxicity of nanoformulation than the free drug (positive control). Thus, it caused the cell viability to decrease by 33%.t In conclusion, PEGylated ethosomal particles had stronger cytotoxicity and longer half-life as compared to the free drug on the cell line of SK-MEL-3 in vitro study [98].

Furthermore, Yu et al. have incorporated the chemotherapeutic agent, mitoxantrone (MTO) into ethosome gel by using a thin-film dispersion method. This was developed to improve the anti-melanoma effect for transdermal melanoma therapy [99]. According to the results of this study, the average size and zeta potential of the MTO ethosomes were 78 ± 4.8 nm and −55 ± 2.6 mV, respectively. The particle was stable as zeta potential had high charges and produced high electrostatic repulsions and mostly were presented as independent vesicles. From the in vitro permeation study, MTO solution showed low permeation ability within 24 h.

On the contrary, MTO ethosome gel demonstrated good permeation of skin which is desirable for melanoma therapy. According to the in vitro study, significant cytotoxic effects on B_16_ melanoma cells with the treatment of MTO ethosome gel were observed along with a rapidly decreasing impendence profile on cellular electrical impedance. This may be due to MTO ethosomes can deliver the drugs transdermally. In contrast, no effects on the B_16_ melanoma cells were observed with blank ethosome gel (negative control) and culture medium. Moreover, based on the immunohistochemical study, calreticulin (CRT) expression on the B_16_ melanoma cells surface is upregulated by MTO ethosome, inducing immune activation that subsequently kills melanoma cells. Thus, therapy of skin melanoma uses MTO ethosome gel as it is an effective transdermal delivery system and potent non-invasion with no serious side effects [99].

In addition, Lin et al. investigated ethosome containing evodiamine (EVO) and Berberine chloride (BBR) [100]. This study was to plan a novel topical anti-melanoma formulation via a single-step injection technique. Different amounts of soybean lecithin (SPC) and propylene glycol (PG) cholesterol also formed together. As the aSPC has increased (180 mg), the particle size of ethosomes also increased (227 ± 6 nm). In this study, ethanol also affected the particle size because it increased the total of alcohol retained inside and in between the layer of vesicular phospholipid. SPC acted as an excipient and deformability property of ethosomes. Therefore, the efficiency of entrapment for EVO and BBR of the ethosomes formulation was above 90%. In the in vitro study, ethosome delivered EVO and BBR into the epidermis. Ethosome also improved permeability and drug delivery. In conclusion, the combination of ethosomes containing EVOO and BBR increased the B_16_ melanoma cells and improved the anti-melanoma effects as a potential delivery system [100].

**Figure 7:**
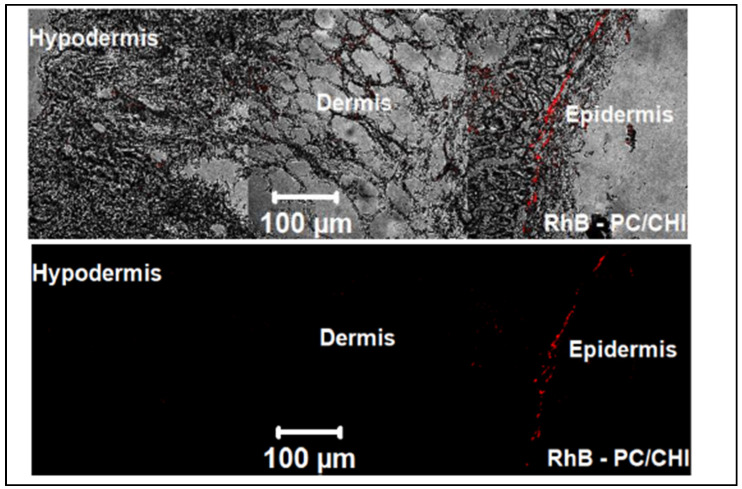
Confocal laser microscopy was used to evaluate the dermal delivery of Fe-CHL loaded ethosomes [101].

Moreover, Nasr et al. prepared ethosomes and lipid-coated chitosan (PC/CHI) containing ferrous chlorophyll (FE-CHL) with a simple modification to treat SCC by photodynamic therapy (PDT) [101]. The confocal laser microscopy and high-performance liquid chromatography penetrated the Fe-CHL depth through mice skin ex vivo and skin retention test. Then, the red fluorescent color was showed the different locations of ethosome and PC/CHI in the skin (Figure 7). The mean vesicle size of ethosome (383 ± 8.1 nm) was larger than PC/CHI (201 ± 5.3 nm), but they showed deeper penetration into the skin. After 24 h, the skin retention percentage of the ethosome had a good result (36% ± 4.5) than FE-CHL solution and coated PC/CHI as 28% ± 3 and 0% and 9% ± 2.8. Confocal laser microscopy showed the skin images of mice which ethosomes had a deeper penetration into the dermis in PC/CHI. PDT effect and 3D spheroids studied the nanocarriers and compared their skin delivery applications on A431 human squamous carcinoma cells. The result of PC/CHI had a higher effection in PDT and 3D spheroids compared to ethosomes. In a nutshell, ethosomes and PC/CHI have the potential to treat SCC by PDT. Additionally, it depends on different locations of tumors in the skin to use it [101].

Apart from that, Moolakkadath et al. prepared binary ethosomes containing fisetin and optimized them by the design of Box–Behnken [102]. The result showed a limited effect on the vesicle size when the phospholipid content increased and ethanol and propylene glycol concentrations were constant. The polynomial equation indicated that efficiency of entrapment had a positive effect because the ethanol showed a mixed effect between the binary ethosomes vesicle and fisetin. Characteristics of fisetin binary ethosome had vesicle size (99.89 ± 3.24 nm), entrapment efficiency (89.23 ± 2.13%), and flux (1.01 ± 0.03 µg/cm^2^/h). Moreover, a negative effect was shown by propylene glycol on the skin when the positive impact was shown by ethanol and phospholipid on flux. Moreover, the rhodamine-B-loaded (control) binary ethosomes formulation had shown the deeper penetration with the confocal images of the rat skin, using confocal laser scanning microscopy. The skin of rats treated in fisetin binary ethosome gel showed an outstanding increase in and AUC_0–8_ and C_skin max_ as compared to the skin of rats treated in fisetin conventional gel. An in vivo study showed a decrease in tumor necrosis factor levels, which are 553 ± 12 pg/mL and 934 ± 15 pg/mL, respectively in the mice pretreated with binary ethosomes gel containing fisetin than to the mice exposed to UV. Furthermore, the percentage of tumor incidence of fisetin binary ethosomes gel in mice (49%) was lower than the mice exposed to UV only (96%). In conclusion, skin cancer can use binary ethosomes containing fisetin formulation to treat, and ethosomes can be the potential dermal delivery system [102].

In the another study, Kollipara et al. prepared curcumin-loaded ethosomes by using a hot method with a slight modification by employing probe sonication [103]. The different formulations’ excipients demonstrated a different variation that affected the formulation characteristics in which the optimized curcumin-loaded ethosomes (F4) showed the zeta potential (−11.9 mV), with the smallest vesicle size (197.7 nm), as well as good polydispersity index (0.34). F4 formulation with ethanol (4.5%), cholesterol (10%), and soya lecithin (10%) were prepared by batch and demonstrated the maximum value was 71.2 ± 3.12 on entrapment efficiency. Based on the permeation studies in vitro, the dialysis bag withdrew the samples simultaneously. From the dialysis bag, the cumulative total of drug released was higher in percentages in ethosomal gel (92.10 ± 2.36%) than the in-house PTX gel (86.26 ± 2.73%) at the end of 12 h. When the time increased, there was a steady increase in the drug concentration in the receptor chamber in the dialysis bag, thus increasing the permeability which followed Fick’s diffusion law. It is proven that the ethosome gel containing curcumin is a better formulation and requires studies in the future. In an ex vivo study, the result showed the curcumin-loaded ethosomal gel showed better treatment than in-house PTX gel in terms of drug deposition on the skin which was >60% in 12 h, whereas in-house PTX gel is shown the drug deposition was <60% in 12 h. In a nutshell, curcumin-loaded ethosomal gel allowed for the retention of curcumin in the deeper skin layers for a prolonged period, which helped completely eradicate the melanoma cells without inducing any painful sensation on the skin [103].

Moreover, Khan and Wong prepared an ethosome containing 5-FU via mechanical dispersion technique with the microwave. As a result, the drug permeation and retention were increased with the skin pretreated with microwave at 2450 MHz for 2.5 min as compared to skin pretreated with ethosome alone. The characterization of ethosome-containing 5-FU revealed that the value of average size (128.9 ± 0.4 nm), polydispersibility index, (0.07 ± 0.01), and average zeta potential (7.6 ± 0.6 mV). This was because microwaves improve skin fluidity and thus improve the skin penetration of the ethosome. The in vitro drug permeability remained unchanged in the skin pretreatment by microwave. The in vivo study revealed that there is a reduction of drug permeation into the blood. This was supported by the reduction of C_max_ and AUC_0–∞_, which were from 12.9 ± 1.1 µg/mL and 188.9 ± 43.8 µg.h/mL to 2.9 ± 0.3 µg/mL and 68.9 ± 11.5 µg.h/mL, respectively, due to the physiological differences between living tissue and dead skin tissue. Furthermore, based on the in vitro cytotoxicity profiles of microwave or ethosomes alone and along with microwave, which MTT assay assessed on the SKMEL-28 human melanoma cells, the combination of microwave and ethosome demonstrated the highest cytotoxicity effect (30%) on pretreated with SKMEL-28 human melanoma cells as compared to others. This was because microwave acts as radiation that targets the tumors residing in melanoma cells and increased the permeability to the deeper region of the skin. In conclusion, the synergistic use of microwave and ethosomes is beneficial in the treatment of skin malignant melanoma (MM) [104].

Based on the studies discussed above, when PTX was loaded into ethosome, it was shown to prolong the drug releases to the target site to kill the cancer cell [98,103]. Moreover, there was a comparison between the curcumin-loaded ethosome and in-house PTX-loaded ethosome in these studies. It showed a greater drug deposition of more than 60% on the skin in curcumin-loaded ethosome, while the drug deposition was less than 60% in in-house PTX loaded ethosome [104,105]. Therefore, ethosomal gel revealed the maximum release in 12 h in ethosomal gel and in-house PTX gel, respectively, in vitro and ex vivo studies. These can also be taken in vitro and in vivo studies for the future. However, it still required further studies for both preclinical and clinical studies to improve the benefit of the prepared ethosomal gel [104].

In conclusion, ethosome is a suitable type of vesicular drug delivery for skin cancer treatment. It improves the drug permeability across the skin and increases the drug loading efficiency. Moreover, the high zeta potential of ethosomes indicates the independent repulsion, and it does not cause aggregation or fusion of the vesicles due to electrostatic repulsion, which resulted in a stable formulation.

In short, several studies have shown the therapeutic benefits of liposomes, niosomes, transfersomes, and ethosomes in the skin cancer treatment via the topical route of administration. The summary of the recent studies of various vesicular drug delivery systems for skin cancer therapy, as discussed earlier, is listed in Table 2.

## 6. Beneficial Aspects of Vesicular Drug Delivery over Another Nanocarrier in Treatment of Skin Cancer

### 6.1. Liposomes

Liposomes received significant interest in biomedicine, especially as an antitumor drug delivery device. It has shown many benefits over traditional systems, not only increased medication utilization and safety from environmental causes of active drug, but also improved product efficiency characteristics, avoiding early drug deterioration, cost-effective drug formulae, and successful therapy with decreased systemic toxicity [105]. In addition, the pharmacokinetic characteristic of liposome products has improved relative to free solution drugs [106]. As a model or carrier for different bioactive agents, such as medications, vaccines, cosmetics, and nutraceuticals, liposomes are widely used. They became perfect medication carriers because of the biocompatible and biodegradable composition of liposomes [107]. Their distinctive ability, correspondingly in their laminae and aqueous core, has augmented their application in the formulations of biomedicine to handle both lipid-soluble and water-soluble agents. Different polymers, PEG and poly (lactic-co-glycolic acid), improve their stability and half-life (PLGA). Polymers, including PEG, have demonstrated an extended half-life of blood circulation.

### 6.2. Niosomes

One of the main components of niosomes included non-ionic surfactants. The surfactant can act as a drug-permeation enhancer by fluidizing the packing of SC lipids [66]. In addition, the types of surfactants used in the formulation can affect the fluidity of the vesicle’s bilayers. Hence, the stability of niosomes can be modified by using different surfactants. Its enhanced stability offered niosomes greater resistance to chemical degradation or oxidation and prolonged storage time [78,83]. Furthermore, the simple methodology for preparing niosomes and its large-scale production without the use of hazardous solvents made it a great alternative to phospholipid-based NPs [79,84,108].

The amphiphilic nature of the surfactant enables the encapsulation of hydrophilic and hydrophobic drugs in the aqueous core and lipophilic domain respectively. The entrapped drug molecules in the niosomes serve as a reservoir that contributes to the sustained release of active substances at a controlled rate [109]. The vesicles also offer protection to drug moiety against the harsh biological environment or first-pass effect [77]. This provides the advantage of improved therapeutic efficacy by increasing drug bioavailability, prolonging half-life, and delaying drug clearance [78]. In addition, surface modification of vesicles enhances the drug targeting ability [78,109]. Ergo, dose reduction of the drug and toxicity risk minimization can be achieved.

### 6.3. Transfersomes

Self-optimization and self-regulating properties of transfersomes allow the vesicle to travel through different transport barriers and help to carry active constituents into target sites with a non-invasive method [84]. In addition, transfersomes have more elastic characteristics than conventional liposomes, due to the aqueous core and lipid bilayers, which allow them to move through narrow sections of the skin that are smaller than vesicle size [84,85]. Having elastic characteristics allows the vesicle to penetrate regions that are smaller than its vesicle diameters. Moreover, the most important component of transfersomes is the edge activators. Edge activators that act as surfactants allow the transfersome to be destabilized, which results in the deformability of the vesicle membrane that allows better permeation when edge activators are combined at proper ratios [86,88]. In addition, transfersome can be used for the entrapment of drugs with different molecular weights, ranging from low to high molecular weight, and can also act as a controlled-released formulation [67]. With the availability of these advantages, transfersome can serve as a potential nanocarrier for drugs in the treatment of skin cancer.

### 6.4. Ethosomes

Ethosomes contain a high ethanol concentration of 20–45%, and ethanol is known as a productive permeation enhancer [96]. Ethanol interacts with the lipid molecule, which has a polar head group region; it reduces the stiffness of the skin and increases fluidity. Thus, ethosome could increase skin permeability. Moreover, ethosome displays as a highly efficient carrier to deliver the drug through the skin for the management of skin cancer [107]. For example, ethosomes act as a drug carrier for various therapeutic agents, such as 5-FU, PTX, mitoxantrone, berberine chloride, evodiamine, ferrous chlorophyll, fisetin, and curcumin [98,99,100,101,102,103,104]. Therefore, ethosome can be an efficient drug delivery in the treatment of skin cancer.

## 7. Safety Concern of Vesicular Drug Delivery for Skin Cancer Treatment and Its Clinical Aspects against Skin Cancer

As discussed in the previous section, most of the vesicular drug delivery for skin cancer treatment appears to be safe, with minimized systemic toxicity, according to the in vitro and preclinical studies. It was found that liposomes are particularly effective in skin cancer therapy. Liposomes can reduce the toxic side effects of chemotherapeutic agents, while improving the effectiveness of antitumor activity, and, thus, they have been regarded as being effective [108]. However, there have been cases of complement activation-related pseudoallergy (CARPA) by using lipid nanoparticles. The pseudoallergy occurs when there is no prior sensitization; hence, the complement system is activated when first exposed to lipid excipients. For example, PEGylated liposomes encapsulating Dox have caused infusion reactions in almost half the cancer patients when they were not given premedications, such as antihistamines and steroids [110]. Meanwhile, the development of niosomes, transfersomes, and ethosomes for the treatment of skin cancer appeared to provide a desirable safety profile with no toxicity issue has been reported.

To date, there is limited information on the topical formulation of vesicular drug delivery systems in the clinical-trial phases, although there was a multitude of in vivo and preclinical studies to support their therapeutic use in skin cancer treatment. Topical drug use has the potential to reduce the local drug concentration and side effects over systematic delivery in dermatological diseases and skin cancer. However, the skin’s stratum corneum is an effective barrier, and its success depends on specific compound significance attached to chemical properties molecular size [111]. A first-class Hedgehog Signaling Action Inhibitor, the Vismodegib, was approved for BCC treatment by the European Medicines Agency (EMA) and by the US Food and Drug Administration (FDA), respectively, in 2012 and 2013. Inadequate activation in many adult tissues of the Hedgehog signaling system is consistent with BCC [112]. Currently, Vismodegib is treated with the use of capsules orally. While the medicinal effects of Vismodegib has been proven during clinical trials, it also causes adverse effects on systemic control. Fatigue, dysgeusia, muscle spasms, nausea, and alopecia were the most common side effects reported in patients receiving Vismodegib. Consequently, formulating Vismodegib in a nano-drug delivery system that could decrease systemic distribution and improve the specific focus of the medicine can improve its effectiveness and thus enable the non-invasive and localized BCC treatment [112]. Therefore, further clinical studies are required to evaluate the vesicular drug delivery’s therapeutic potentials, safety, and effectiveness against skin cancer.

## 8. Conclusions and Future Perspectives

Globally, millions of people, especially Caucasians, are affected by skin cancer every year. Although skin cancer is not the leading cause of mortality among all types of cancer, the significant impact of skin cancer on the economic burden, patients’ QoL, and limitations in the current treatments available justify the necessity of an effective and safe novel treatment option for skin cancer. Topical drug delivery offers less invasive routes of administration, provides convenience to patients, and minimizes life-threatening side effects, as compared to systemic chemotherapy. However, several drawbacks from the current topical treatments, such as poor skin penetration, frequent and prolonged applications, severe skin irritation when topically applied over large skin areas, and non-targeted drug delivery, have led to poor patient adherence to therapy.

Among all the treatment approaches studied in the current research, nanotechnology has received much attention in terms of skin cancer treatment. The vesicular drug delivery system has been widely investigated and evolved to be an important alternative to the existing drug delivery strategy in treating skin cancer, especially melanoma. This is due to their nanosize and ability to improve the penetration of the anticancer drug through the skin, reaching the cancer site specifically with sufficient levels and achieving prolonged retention at the tumor site to kill the cancer cells. Moreover, the vesicles can protect the encapsulated drug from degradation and exhibit low skin-irritation potential. As a result, treatment efficacy is greatly improved, and unwanted systemic toxicity effects are prevented or minimized. Subsequently, the skin-cancer patients’ survival and treatment adherence is enhanced.

Liposomes, the most well-established lipid vesicles, are shown to provide various advantages in topical drug delivery. For instance, a drug carrier for hydrophilic and lipophilic drugs; bio-inert, biodegradable, controlled release of therapeutic agents; and its lipid vesicles composition that is highly similar to those of epidermis, allowing greater extent of penetration across the epidermal barrier, and act as a reservoir for localized action. It has been proven that liposomes can effectively transport anticancer drugs to cancer cells and increase the efficiency of the therapeutic agent. Other than that, the ultra-deformable lipid vesicles, namely the ethosomes, niosomes, and transfersomes, have been developed to enhance further the skin permeation of the encapsulated drugs in the conventional liposomes. The presence of non-ionic surfactants in niosomes loosens the SC layer and enhances skin permeability. The niosomal nanocarrier also showed an enhancement of the drug efficacy and targeting, as well as minimization of toxicity risk. On the other hand, transfersomes contain an edge activator that enables the vesicles to squeeze through the intercellular space of SC, while the ethosomes have a relatively high ethanol concentration that improves their skin-penetration property.

Given the potential benefits of the vesicular drug delivery system, various studies have been performed to examine their potential topical application in skin cancer treatment. Promising results have been obtained from the in vitro and preclinical studies on the topical skin delivery of drugs and bioactive agents over the years. Moreover, these vesicular drug delivery systems could offer potentially cost-effective, safe, and therapeutic effective treatment options to skin cancer patients compared to more expensive conventional treatments, such as gene therapies and immunotherapies. This leads to the need of translating these nanocarrier systems into clinical applications. However, several factors would need to be addressed and investigated, such as the long-term toxicity and degradation, clinical pharmacokinetics, the long-term and short-term impact of repeated applications of the formulations on the skin, and inter-individual variation in skin cancer conditions and response towards the treatment. Moreover, it is crucial to select the most suitable component for topical formulation development for skin cancer treatment and simplify the drug delivery design to enable large-scale production for commercial development and meet the regulatory requirement.

In short, the vesicular drug delivery system is a promising strategy to be utilized in the treatment of skin cancer. Thus, many efforts and investigations on their therapeutic applications for skin cancer treatment are still required to develop an optimal, individualized, effective, and safe treatment for skin cancer patients.

## Figures and Tables

**Table 1:** Summary of recent studies of nanocarriers for skin cancer therapy.

Objectives	Type of Nanocarriers	Polymer Used	Drug	Cell Line/Animal Model	Outcomes	Source
To compare the effect of sodium alginate and chitosan on NE in terms of penetration-enhancing effects.	Nano emulsion	Chitosan or sodium alginate	Piplartine (piperlongumine)	2D cell cultures of melanoma cells (SK-MEL-28)	Chitosan- and alginate-modified NE enhanced skin penetration and higher cytotoxic effect of piplartine.	[50]
To prepare, optimize, and compare the effects of 5-FU–NE and carbopol based 5-FU–NE-Gel on melanoma cell lines and determine the retention and penetration of 5-FU using cow, goat, and rat skin models.	Nano emulsions Nano emulsion gel	Carbopol 934	5-FU	Melanoma cancer cell lines (SK-MEL-5-type) Swiss albino rat full-thickness skins Ear pinna skin from goat and cow	Demonstrated smallest globule size, viscosity, refractive index, and polydispersity index value with maximum droplet size uniformity and optimum zeta potential. Moreover, 5-FU–NE3-Gel and optimized-5-FU–NE3 showed significantly higher cytotoxic effect and permeation than 5-FU-S.	[53]
To engineer 5-FU encapsulated biodegradable chitosan nanogels for topical chemotherapy.	Nanogel	Chitosan Pluronic F-127	5-FU	Human keratinocyte (HaCaT) cell line Swiss albino male mice (DMBA induced melanoma mice model)	The engineered 5-FU-loaded, pH-responsive, and biocompatible nanogel provides immediate burst release, followed by slow and sustained drug release in the acidic melanoma tumor microenvironment with reduced side effects.	[54]
To study Apatinib-loaded NP on the inhibition of tumor growth and angiogenesis in melanoma model.	Synthetic polymeric nanoparticle	PLGA	Apatinib	Tumor B16 cells Mouse melanoma model	Drug-loaded nanoparticles reduced the growth of tumor cells with a high cytotoxic effect on tumor B16 cells.	[56]
To overcome the potential challenge through a nanofibrous scaffold by localizing MoO3 nanoparticles.	Nanofiber	Polycaprolactone	Molybdenum trioxide	Zebra fish	Enhanced targeted delivery of anticancer drug to treat skin cancer.	[58]
To treat skin cancer non-invasively using an external alternating current (AC) magnetic field-induced hyperthermia.	Nanofiber	Polycaprolactone	Iron Oxide	Hela cells and BALB/c mice	Skin cancer was treated by confirming the PCL-Fe3O4 nanofibrous-based bandages are sole and compelling.	[59]
To study the effect of layer-by-layer polymer-coated gold nanoparticles (AuNP) for topical delivery of imatinib mesylate (IM) in the treatment of melanoma.	Gold nanoparticles	Anionic poly(styrenesulfonate), cationic polyethylene imine	Imatinib mesylate	B16F10 melanoma cells porcine ear skin	Metal nanoparticles showed enhanced skin permeation and cytotoxicity against melanoma cells.	[61]
To investigate the use of superparamagnetic iron oxide NP as transdermal drug delivery carrier for epirubicin (EPI) in the treatment of skin cancer.	Superparamagnetic iron oxide nanoparticles		Epirubicin	WM266 melanoma cells	Improve skin permeation by using external magnetic force, and pH-responsive drug-release pattern allows the targeted delivery.	[62]
To evaluate the ability of SLN to deliver 5-FU via the skin.	Solid lipid nanoparticle	Lecithin, poloxamer 188	5-FU	BALB/c (Bagg albino) mice	SLN formula can penetrate lipophilic membranes to a greater extent than the free drug and enhance the effects of the drug.	[64]
To investigate the use of sesamol-loaded SLN in a topical cream for the treatment of skin cancer.	Solid lipid nanoparticle	Glyceryl monostearate	Sesamol	Molt-4 and HL-60 cancer cell lines LACA mice	The onset of tumors was delayed when they were treated with sesamol and SLN, due to apoptotic cell death.	[65]

**Table 2:** Summary of recent studies of various vesicular drug delivery systems for skin cancer therapy.

Objectives	Type of Nanocarriers	Polymer Used	Drug	Cell Line/ Animal Model	Outcomes	Source
To develop a liposomal melanoma target-delivery system that co-delivers tumor necrosis factor-related apoptosis-inducing ligand (TRAIL) and paclitaxel (PTX) against melanoma.	Liposomes	Soybean lecithin (S100), cholesterol, DSPE-PEG2000	TRAIL and Paclitaxel	B16F10 (mouse melanoma cell line) MCF-7 cells (human breast cancer cell line) Female C57 BL/6 mice	Liposomes improved stability and drug release profile along with selective delivery to tumorous cells. Significantly improved drug biodistribution and anticancer efficiency in tumor-bearing mice.	[73]
To develop an EGFR-targeted immunoliposome loaded with 5-FU to allow co-administration of the antibody and the chemotherapeutic agent and achieve selective delivery to SCC.	Liposomes	DSPC, cholesterol, DSPE-PEG-Mal	5-FU, cetuximab	SCC xenograft animal model EGFR-positive SCC cells Porcine ear skin	The absorption and penetration of immunoliposomes were higher compared to liposomes. Immunoliposomes had smaller tumors after iontophoresis administration compared to 5-FU solution.	[74]
To develop a peptide-modified vemurafenib-loaded liposome for the targeted inhibition of subcutaneous melanoma via the skin.	Liposomes	DSPE-PEG-NHS, cholesterol, lecithin	Vemurafenib	Human A375 melanoma cells Murine B16F10 melanoma cells Human umbilical vein endothelial cells (HUVEC) Male BALB/c mice (Bagg albino mouse)	Liposomes were successfully internalized by A375 cells with selective inhibition of cancer cells by Vem. Liposomes showed desired antitumor ability at a lower concentration.	[75]
To develop a topical siRNA delivery system that can permeate through the stratum corneum and viable epidermis and efficiently deposit therapeutic levels of siRNA to the basal epidermis/upper dermis where melanoma cells reside.	Liposomes	DOTAP	BRAF siRNA	Human cadaver skin UACC-903 melanoma cells	Liposomes with an 8:1 ratio of DOTAP:NaChol and complexed with siRNA at 16:1 showed the most effective skin permeation rate and significant deposition at upper dermis with higher internalized by melanoma cells.	[76]
To investigate the use of niosomes as topical delivery systems for the treatment of skin cancer with 5-FU.	Niosomes	Cholesterol, α,ω-hexadecyl-bis-(1-aza-18-crown-6), Span 80	5-FU	SKMEL-26 (human melanoma cell) HaCaT (human epidermal keratinocytes)	Niosomes increased percutaneous permeation (8-fold) and anticancer activity.	[80]
To study the anti-melanoma activity of artemisone in niosomal formulation.	Niosomes	Span 60, cholesterol	Artemisone	A-375 (human malignant melanoma cell) HaCaT (human epidermal keratinocytes)	Niosomes increase anticancer activity with negligible toxicity against normal skin.	[82]
To investigate improving skin absorption of 5-FU for treatment of actinic keratosis and non-melanoma skin cancer.	Transfersome	PC, Tween-80, Span-80	5-FU	Dorsal skin of mice (Swiss albino male mice)	Transfersomal gel showed better entrapment and drug deposition.	[87]
To study skin cancer prevention by using carvedilol loaded transfersomes.	Transfersome	SPC, HEPC, DSPC, Tween-80, sodium cholate	Carvedilol	Porcine ear skin Mouse epidermal cell line 3D Human Reconstituted Skin Model	Drug permeation for transfersome was lower than a free drug with a photoprotective effect.	[89]
To study on treatment of melanoma skin cancer by using paclitaxel loaded transfersomes.	Transfersome	PC, Span-80	Paclitaxel		Transfersome showed the highest entrapment efficacy and the highest percentage of drug released.	[91]
To study on tocopherol-loaded transfersome for evaluation of antioxidant and skin regenerative properties.	Transfersome	Soy PC, alpha-tocopherol acetate, Tween-20, Tween-40, Tween-60, Tween-80	Alpha-tocopherol	One-day old pigs’ dorsal skin Human epidermal keratinocytes Mouse embryonic fibroblast	Transfersome using Tween-80 showed the highest entrapment efficiency and smallest vesicle size with antioxidant effect.	[93]
To investigate topical photodynamic therapy by using transfersomal AlPcS4.	Transfersome	PC, Sodium deoxycholate	AlPcS4	Baby hamster kidney (BHK)-21 fibroblasts cell line BALB/c mice’s dorsal skin	AlPcS4-loaded transfersome showed better uptake into the skin and deeper penetration.	[94]
To study and feature ethosome particles containing Paclitaxel^®^ and nano-drug is compared the efficacy to the free drug on the cell line of human melanoma SK-MEL-3.	Ethosome	Polyethylene glycol, cholesterol	Paclitaxel	SK-MEL-3 (Human melanoma cell)	PEGylated ethosomes increased the encapsulation efficiency of drug loading and decrease the cell viability of tumor cells.	[98]
To improve the anti-melanoma effect of a transdermal mitoxantrone ethosome gel.	Ethosome	Gel, soybean phospholipid	Mitoxantrone	B16 melanoma cells BALB/c nude nice	Improve permeability and cytotoxic effect of MTO with ethosome. Calreticulin expression was improved by the MTO ethosome gel on B16 melanoma cells.	[99]
To modify an anti-melanoma function of novel topical.	Ethosome	Propylene glycol, soybean lecithin, cholesterol	Berberine chloride, evodiamine	B16 melanoma cell	Improved skin permeability and drug delivery. Anti-melanoma effects were improved on B16 melanoma cells.	[100]
To investigate FE-CHI loaded in both PC/CHI nanocarrier and ethosomes comparing their skin delivery applications and PDT effect.	Ethosome	Polyhydroxyethylmethacrylate, soya lecithin	Ferrous chlorophyll	A431 human epithelial squamous carcinoma cell, The skin of albino mice	The entrapment efficiency of EVO and BBR of the ethosomes formulation enhanced with decrease the levels of TNF-α and IL-1α in ethosome gel treated mice.	[101]
To assess the efficacy of binary ethosomes containing fisetin formulation for skin cancer management in models of the animal.	Ethosome	Propylene glycol, phospholipid, diethyl ester	Fisetin	Swiss albino mice	Mice skin treated with ethosome gel showed an increase in AUC0-8 and C skin max with decreased levels of TNF-α and IL-1α.	[102]
To study the treatment of skin melanoma in formulating and evaluating the curcumin-loaded ethosomes to enhance the solubility and permeability for skin melanomas’ treatment.	Ethosome	Polystyrene, cholesterol, soya lecithin	Curcumin	Rat dorsal ear skin	Ethosome gel containing Curcumin showed better release and drug deposition. Curcumin-loaded ethosome gel allows retention of curcumin in the deeper skin to completely eradicate the melanoma cells.	[103]
To promote penetration of the skin and/or deposition of 5-FU in vitro and in vivo.	Unilamellar Ethosome	Soya phosphotidylcholine	5-FU	SKMEL-28 human melanoma cell Male Sprague Dawley rat	The combination of microwave and ethosome demonstrated the significant cytotoxicity effect on SKMEL-28 cells with increased retention in the skin.	[104]

## Data Availability

Data are freely available.

## References

[1] Yousef H., Sharma S. (2020). Anatomy, Skin (Integument), Epidermis.

[2] Akhtar N., Khan R.A. (2016). Liposomal systems as viable drug delivery technology for skin cancer sites with an outlook on lipid-based delivery vehicles and diagnostic imaging inputs for skin conditions’. Prog. Lipid Res..

[3] Khazaei Z., Ghorat F., Jarrahi A.M., Adineh H.A., Sohrabivafa M., Goodarzi E. (2019). Global incidence and mortality of skin cancer by histological subtype and its relationship with the human development index (HDI); An ecology study in 2018. World Cancer Res. J..

[4] Narayanan D.L., Saladi R.N., Fox J.L. (2010). Ultraviolet radiation and skin cancer. Int. J. Dermatol..

[5] Radiation: Ultraviolet (UV) Radiation and Skin Cancer. https://www.who.int/news-room/q-a-detail/radiation-ultraviolet-(uv)-radiation-and-skin-cancer.

[6] Affandi A.M. (2018). Skin cancer: 13-year experience at the Department of Dermatology, Hospital Kuala Lumpur, Malaysia. J. Glob. Oncol..

[7] International Agency for Research on Cancer (2020). Malaysia|Source: Globocan 2020.

[8] Guy G.P., Machlin S.R., Ekwueme D.U., Yabroff K.R. (2015). Prevalence and costs of skin cancer treatment in the U.S., 2002–2006 and 2007–2011. Am. J. Prev. Med..

[9] Linares M.A., Zakaria A., Nizran P. (2015). Skin Cancer. Prim. Care Clin. Off. Pract..

[10] Simões M.C.F., Sousa J.J.S., Pais A.A.C.C. (2015). Skin cancer and new treatment perspectives: A review. Cancer Lett..

[11] Losquadro W.D. (2017). Anatomy of the skin and the pathogenesis of nonmelanoma skin cancer. Facial Plast. Surg. Clin. N. Am..

[12] Krishnan V., Mitragotri S. (2020). Nanoparticles for topical drug delivery: Potential for skin cancer treatment. Adv. Drug Deliv. Rev..

[13] International Agency for Research on Cancer (2020). Non-Melanoma Skin Cancer|Source: Globocan 2020.

[14] Bray F., Ferlay J., Soerjomataram I., Siegel R.L., Torre L.A., Jemal A. (2018). Global cancer statistics 2018: GLOBOCAN estimates of incidence and mortality worldwide for 36 cancers in 185 countries. CA Cancer J. Clin..

[15] Didona D., Paolino G., Bottoni U., Cantisani C. (2018). Non melanoma skin cancer pathogenesis overview. Biomedicines.

[16] Cameron M.C., Lee E., Hibler B.P., Barker C.A., Mori S., Cordova M., Nehal K.S., Rossi A.M. (2019). Basal cell carcinoma: Epidemiology; pathophysiology; clinical and histological subtypes; and disease associations. J. Am. Acad. Dermatol..

[17] Fania L., Didona D., Morese R., Campana I., Coco V., Di Pietro F.R., Ricci F., Pallotta S., Candi E., Abeni D. (2020). Basal cell carcinoma: From pathophysiology to novel therapeutic approaches. Biomedicines.

[18] Skin Cancer Foundation Melanoma Overview. https://www.skincancer.org/skin-cancer-information/melanoma/.

[19] Orthaber K., Pristovnik M., Skok K., Perić B., Maver U. (2017). Skin cancer and its treatment: Novel treatment approaches with emphasis on nanotechnology. J. Nanomater..

[20] International Agency for Research on Cancer (2020). World|Source: Globocan 2020.

[21] Oliveria S.A., Saraiya M., Geller A.C., Heneghan M.K., Jorgensen C. (2006). Sun exposure and risk of melanoma. Arch. Dis. Child..

[22] Kato J., Horimoto K., Sato S., Minowa T., Uhara H. (2019). Dermoscopy of melanoma and non-melanoma skin cancers. Front. Med..

[23] Ismail M., Khan S., Khan F., Noor S., Sajid H., Yar S., Rasheed I. (2020). Prevalence and significance of potential drug-drug interactions among cancer patients receiving chemotherapy. BMC Cancer.

[24] Xiao H., Noble G.T., Stefanick J.F., Qi R., Kiziltepe T., Jing X., Bilgicer B. (2014). Photosensitive Pt(IV)-azide prodrug-loaded nanoparticles exhibit controlled drug release and enhanced efficacy in vivo. J. Control Release.

[25] Mou Q., Ma Y., Zhu X., Yan D. (2016). A small molecule nanodrug consisting of amphiphilic targeting ligand-chemotherapy drug conjugate for targeted cancer therapy. J. Control Release.

[26] Buajordet I., Ebbesen J., Erikssen J., Brørs O., Hilberg T. (2001). Fatal adverse drug events: The paradox of drug treatment. J. Intern. Med..

[27] Neville J.A., Welch E., Leffell D.J. (2007). Management of nonmelanoma skin cancer in 2007. Nat. Clin. Pract. Oncol..

[28] Chakrabarty A., Geisse J.K. (2004). Medical therapies for non-melanoma skin cancer. Clin. Dermatol..

[29] Marks R., Gebauer K., Shumack S., Amies M., Bryden J., Fox T.L., Owens M.L. (2001). Imiquimod 5% cream in the treatment of superficial basal cell carcinoma: Results of a multicenter 6-week dose-response trial. J. Am. Acad. Dermatol..

[30] Shumack S., Robinson J., Kossard S., Golitz L., Greenway H., Schroeter A., Andres K., Amies M., Owens M. (2002). Efficacy of topical 5% imiquimod cream for the treatment of nodular basal cell carcinoma. Arch. Dermatol..

[31] Chua B., Jackson J.E., Lin C., Veness M.J. (2019). Radiotherapy for early non-melanoma skin cancer. Oral Oncol..

[32] Chen E.L.A., Srivastava D., Nijhawan R.I. (2018). Mohs micrographic surgery: Development, technique, and applications in cutaneous malignancies. Semin. Plast. Surg..

[33] Wain R.A.J., Tehrani H. (2015). Reconstructive outcomes of Mohs surgery compared with conventional excision: A 13-month prospective study. Br. J. Plast. Surg..

[34] Downes R.N., Walker N.P.J., Collin J.R.O. (1990). Micrographic (MOHS’) surgery in the management of periocular basal cell epitheliomas. Eye.

[35] Gorzelanny C., Mess C., Schneider S.W., Huck V., Brandner J.M. (2020). Skin barriers in dermal drug delivery: Which barriers have to be overcome and how can we measure them?. Pharmaceutics.

[36] Lee A.Y. (2020). Molecular mechanism of epidermal barrier dysfunction as primary abnormalities. Int. J. Mol. Sci..

[37] Yokouchi M., Kubo A. (2018). Maintenance of tight junction barrier integrity in cell turnover and skin diseases. Exp. Dermatol..

[38] Matsui T., Amagai M. (2015). Dissecting the formation, structure and barrier function of the stratum corneum. Int. Immunol..

[39] Bouwstra J.A., Gooris G.S., van der Spek J.A., Bras W. (1991). Structural investigations of human stratum corneum by small-angle X-ray scattering. J. Investig. Dermatol..

[40] Bouwstra J.A., Gooris G.S., Bras W., Downing D.T. (1995). Lipid organization in pig stratum corneum. J. Lipid Res..

[41] Rancan F., Giulbudagian M., Jurisch J., Blume-Peytavi U., Calderón M., Vogt A. (2017). Drug delivery across intact and disrupted skin barrier: Identification of cell populations interacting with penetrated thermoresponsive nanogels. Eur. J. Pharm. Biopharm..

[42] Kirschner N., Rosenthal R., Furuse M., Moll I., Fromm M., Brandner J.M. (2013). Contribution of tight junction proteins to ion, macromolecule, and water barrier in keratinocytes. J. Investig. Dermatol..

[43] Brandner J.M., Zorn-Kruppa M., Yoshida T., Moll I., Beck L.A., De Benedetto A. (2015). Epidermal tight junctions in health and disease. Tissue Barriers.

[44] Oh J.W., Kloepper J., Langan E.A., Kim Y., Yeo J., Kim M.J., Hsi T.C., Rose C., Yoon G.S., Lee S.J. (2016). A guide to studying human hair follicle cycling in vivo. J. Investig. Dermatol..

[45] Zorn-Kruppa M., Vidal-y-Sy S., Houdek P., Wladykowski E., Grzybowski S., Gruber R., Gorzelanny C., Harcup J., Schneider S.W., Majumdar A. (2018). Tight Junction barriers in human hair follicles—Role of claudin-1. Sci. Rep..

[46] Petrofsky J.S. (2017). Control of skin Blood Flow. Textbook of Aging Skin.

[47] Severino P., Fangueiro J.F., Ferreira S.V., Basso R., Chaud M.V., Santana M.H.A., Rosmaninho A., Souto E.B. (2013). Nanoemulsions and nanoparticles for non-melanoma skin cancer: Effects of lipid materials. Clin. Transl. Oncol..

[48] Prabhakar U., Maeda H., Jain R.K., Sevick-Muraca E.M., Zamboni W., Farokhzad O.C., Barry S.T., Gabizon A., Grodzinski P., Blakey D.C. (2013). Challenges and key considerations of the enhanced permeability and retention effect for nanomedicine drug delivery in oncology. Cancer Res..

[49] Maeda H., Greish K., Fang J. (2006). The EPR effect and polymeric drugs: A paradigm shift for cancer chemotherapy in the 21st century. Adv. Polym. Sci..

[50] Giacone D.V., Dartora V.F.M.C., de Matos J.K.R., Passos J.S., Miranda D.A.G., de Oliveira E.A., Silveira E.R., Costa-Lotufo L.V., Maria-Engler S.S., Lopes L.B. (2020). Effect of nanoemulsion modification with chitosan and sodium alginate on the topical delivery and efficacy of the cytotoxic agent piplartine in 2D and 3D skin cancer models. Int. J. Biol. Macromol..

[51] Fofaria N.M., Qhattal H.S.S., Liu X., Srivastava S.K. (2016). Nanoemulsion formulations for anti-cancer agent piplartine—Characterization, toxicological, pharmacokinetics and efficacy studies. Int. J. Pharm..

[52] Entezar-Almahdi E., Mohammadi-Samani S., Tayebi L., Farjadian F. (2020). Recent advances in designing 5-Fluorouracil delivery systems: A stepping stone in the safe treatment of colorectal cancer. Int. J. Nanomed..

[53] Ahmad N., Ahmad R., Mohammed Buheazaha T., Salman AlHomoud H., Al-Nasif H.A., Sarafroz M. (2020). A comparative ex vivo permeation evaluation of a novel 5-Fluorocuracil nanoemulsion-gel by topically applied in the different excised rat, goat, and cow skin. Saudi J. Biol. Sci..

[54] Sahu P., Kashaw S.K., Sau S., Kushwah V., Jain S., Agrawal R.K., Iyer A.K. (2019). pH responsive 5-Fluorouracil loaded biocompatible nanogels for topical chemotherapy of aggressive melanoma. Colloids Surf. B Biointerfaces.

[55] Sahu P., Kashaw S.K., Sau S., Iyer A.K. (2017). Stumuli-responsive bio-hybrid nanogels: An emerging platform in medicinal arena. Glob. J. Nanomed..

[56] Zhang J., Liu P., Zhang Z., Han J., Yang X., Wang A., Zhang X. (2020). Apatinib-loaded nanoparticles inhibit tumor growth and angiogenesis in a model of melanoma. Biochem. Biophys. Res. Commun..

[57] Nanofiber—An Overview|ScienceDirect Topics. https://www.sciencedirect.com/topics/materials-science/nanofibers.

[58] Janani I., Lakra R., Kiran M.S., Korrapati P.S. (2018). Selectivity and sensitivity of molybdenum oxide-polycaprolactone nanofiber composites on skin cancer: Preliminary in-vitro and in-vivo implications. J. Trace Elem. Med. Biol..

[59] Suneet K., De T., Rangarajan A., Jain S. (2020). Magnetic nanofibers based bandage for skin cancer treatment: A non-invasive hyperthermia therapy. Cancer Rep..

[60] Dianzani C., Zara G.P., Maina G., Pettazzoni P., Pizzimenti S., Rossi F., Gigliotti C.L., Ciamporcero E.S., Daga M., Barrera G. (2014). Drug delivery nanoparticles in skin cancers. BioMed Res. Int..

[61] Labala S., Mandapalli P.K., Kurumaddali A., Venuganti V.V.K. (2015). Layer-by-layer polymer coated gold nanoparticles for topical delivery of imatinib mesylate to treat melanoma. Mol. Pharm..

[62] Rao Y.F., Chen W., Liang X.G., Huang Y.Z., Miao J., Liu L., Lou Y., Zhang X.G., Wang B., Tang R.K. (2015). Epirubicin-loaded superparamagnetic iron-oxide nanoparticles for transdermal delivery: Cancer therapy by circumventing the skin barrier. Small.

[63] Bayón-Cordero L., Alkorta I., Arana L. (2019). Application of solid lipid nanoparticles to improve the efficiency of anticancer drugs. Nanomaterials.

[64] Khallaf R.A., Salem H.F., Abdelbary A. (2016). 5-Fluorouracil shell-enriched solid lipid nanoparticles (SLN) for effective skin carcinoma treatment. Drug Deliv..

[65] Geetha T., Kapila M., Prakash O., Deol P.K., Kakkar V., Kaur I.P. (2015). Sesamol-loaded solid lipid nanoparticles for treatment of skin cancer. J. Drug Target..

[66] Hua S. (2015). Lipid-based nano-delivery systems for skin delivery of drugs and bioactives. Front. Pharmacol..

[67] Jain S., Jain V., Mahajan S.C. (2014). Lipid based vesicular drug delivery systems. Adv. Pharm..

[68] Saeed M., Zalba S., Seynhaeve A.L.B., Debets R., Ten Hagen T.L.M. (2019). Liposomes targeted to MHC-restricted antigen improve drug delivery and antimelanoma response. Int. J. Nanomed..

[69] Lamichhane N., Udayakumar T.S., D’Souza W.D., Simone C.B., Raghavan S.R., Polf J., Mahmood J. (2018). Liposomes: Clinical applications and potential for image-guided drug delivery. Molecules.

[70] Rata D.M., Cadinoiu A.N., Atanase L.I., Popa M., Mihai C.T., Solcan C., Ochiuz L., Vochita G. (2021). Topical formulations containing aptamer-functionalized nanocapsules loaded with 5-fluorouracil—An innovative concept for the skin cancer therapy. Mater. Sci. Eng. C.

[71] Choi F.D., Kraus C.N., Elsensohn A.N., Carley S.K., Lehmer L.M., Nguyen R.T., Linden K.G., Shiu J. (2020). Programmed cell death 1 protein and programmed death-ligand 1 inhibitors in the treatment of nonmelanoma skin cancer: A systematic review. J. Am. Acad. Dermatol..

[72] Capanema N.S.V., Mansur A.A.P., Carvalho S.M., Carvalho I.C., Chagas P., de Oliveira L.C.A., Mansur H.S. (2018). Bioengineered carboxymethyl cellulose-doxorubicin prodrug hydrogels for topical chemotherapy of melanoma skin cancer. Carbohydr. Polym..

[73] Huang S., Zhang Y., Wang L., Liu W., Xiao L., Lin Q., Gong T., Sun X., He Q., Zhang Z. (2020). Improved melanoma suppression with target-delivered TRAIL and Paclitaxel by a multifunctional nanocarrier. J. Control Release.

[74] Petrilli R., Eloy J.O., Saggioro F.P., Chesca D.L., de Souza M.C., Dias M.V.S., DaSilva L.L.P., Lee R.J., Lopez R.F.V. (2018). Skin cancer treatment effectiveness is improved by iontophoresis of EGFR-targeted liposomes containing 5-FU compared with subcutaneous injection. J. Control Release.

[75] Zou L., Ding W., Zhang Y., Cheng S., Li F., Ruan R., Wei P., Qiu B. (2018). Peptide-modified vemurafenib-loaded liposomes for targeted inhibition of melanoma via the skin. Biomaterials.

[76] Dorrani M., Garbuzenko O.B., Minko T., Michniak-Kohn B. (2016). Development of edge-activated liposomes for siRNA delivery to human basal epidermis for melanoma therapy. J. Control Release.

[77] Yeo P.L., Lim C.L., Chye S.M., Ling A.P.K., Koh R.Y. (2017). Niosomes: A review of their structure, properties, methods of preparation, and medical applications. Asian Biomed..

[78] Ge X., Wei M., He S., Yuan W.-E. (2019). Advances of non-ionic surfactant vesicles (niosomes) and their application in drug delivery. Pharmaceutics.

[79] Wang J., Sui M., Fan W. (2010). Nanoparticles for tumor targeted therapies and their pharmacokinetics. Curr. Drug Metab..

[80] Paolino D., Cosco D., Muzzalupo R., Trapasso E., Picci N., Fresta M. (2008). Innovative bola-surfactant niosomes as topical delivery systems of 5-fluorouracil for the treatment of skin cancer. Int. J. Pharm..

[81] Aderibigbe B.A. (2017). Design of drug delivery systems containing artemisinin and its derivatives. Molecules.

[82] Dwivedi A., Mazumder A., du Plessis L., du Preez J.L., Haynes R.K., du Plessis J. (2015). In vitro anti-cancer effects of artemisone nano-vesicular formulations on melanoma cells. Nanomed. Nanotechnol. Biol. Med..

[83] Mohamed H.B., El-Shanawany S.M., Hamad M.A., Elsabahy M. (2017). Niosomes: A strategy toward prevention of clinically significant drug incompatibilities. Sci. Rep..

[84] Bhasin B., Londhe V.Y. (2018). An overview of transferosomal drug delivery. Int. J. Pharm. Sci. Res..

[85] Opatha S.A.T., Titapiwatanakun V., Chutoprapat R. (2020). Transfersomes: A promising nanoencapsulation technique for transdermal drug delivery. Pharmaceutics.

[86] Lei W., Yu C., Lin H., Zhou X. (2013). Development of tacrolimus-loaded transfersomes for deeper skin penetration enhancement and therapeutic effect improvement in vivo. Asian J. Pharm. Sci..

[87] Khan M.A., Pandit J., Sultana Y., Sultana S., Ali A., Aqil M., Chauhan M. (2015). Novel carbopol-based transfersomal gel of 5-fluorouracil for skin cancer treatment: In vitro characterization and in vivo study. Drug Deliv..

[88] Pandey A., Mittal A., Chauhan N., Alam S. (2014). Role of surfactants as penetration enhancer in transdermal drug delivery system. J. Mol. Pharm. Org. Process Res..

[89] Chen M., Shamim M.A., Shahid A., Yeung S., Andresen B.T., Wang J., Nekkanti V., Meyskens F.L., Kelly K.M., Huang Y. (2020). Topical delivery of carvedilol loaded nano-transfersomes for skin cancer chemoprevention. Pharmaceutics.

[90] Danaei M., Dehghankhold M., Ataei S., Hasanzadeh Davarani F., Javanmard R., Dokhani A., Khorasani S., Mozafari M. (2018). Impact of particle size and polydispersity index on the clinical applications of lipidic nanocarrier systems. Pharmaceutics.

[91] Raahulan S., Sanapalli B.K.R., Karri V.V.S.R. (2019). Paclitaxel loaded transfersomal vesicular drug delivery for the treatment of melanoma skin cancers. Int. J. Res. Pharm. Sci..

[92] Godic A., Poljšak B., Adamic M., Dahmane R. (2014). The role of antioxidants in skin cancer prevention and treatment. Oxid. Med. Cell. Longev..

[93] Caddeo C., Manca M.L., Peris J.E., Usach I., Diez-Sales O., Matos M., Fernàndez-Busquets X., Fadda A.M., Manconi M. (2018). Tocopherol-loaded transfersomes: In vitro antioxidant activity and efficacy in skin regeneration. Int. J. Pharm..

[94] Kassab K., El Fadeel D.A., Fadel M. (2013). Topical photodynamic therapy using transfersomal aluminum phthalocyanine tetrasulfonate: In vitro and in vivo study. Lasers Med. Sci..

[95] Montanari J., Perez A.P., Di Salvo F., Diz V., Barnadas R., Dicelio L., Doctorovich F., Morilla M.J., Romero E.L. (2007). Photodynamic ultradeformable liposomes: Design and characterization. Int. J. Pharm..

[96] Verma P., Pathak K. (2010). Therapeutic and cosmeceutical potential of ethosomes: An overview. J. Adv. Pharm. Technol. Res..

[97] Garg V., Singh H., Bimbrawh S., Singh S.K., Gulati M., Vaidya Y., Kaur P. (2016). Ethosomes and transfersomes: Principles, perspectives and practices. Curr. Drug Deliv..

[98] Eskolaky E.B., Ardjmand M., Akbarzadeh A. (2015). Evaluation of anti-cancer properties of pegylated ethosomal paclitaxel on human melanoma cell line SKMEL-3. Trop. J. Pharm. Res..

[99] Yu X., Du L., Li Y., Fu G., Jin Y. (2015). Improved anti-melanoma effect of a transdermal mitoxantrone ethosome gel. Biomed. Pharmacother..

[100] Lin H., Lin L., Choi Y., Michniak-Kohn B. (2020). Development and in-vitro evaluation of co-loaded berberine chloride and evodiamine ethosomes for treatment of melanoma. Int. J. Pharm..

[101] Nasr S., Rady M., Gomaa I., Syrovet T., Simmet T., Fayad W., Abdel-Kader M. (2019). Ethosomes and lipid-coated chitosan nanocarriers for skin delivery of a chlorophyll derivative: A potential treatment of squamous cell carcinoma by photodynamic therapy. Int. J. Pharm..

[102] Moolakkadath T., Aqil M., Ahad A., Imam S.S., Praveen A., Sultana Y., Mujeeb M., Iqbal Z. (2019). Fisetin loaded binary ethosomes for management of skin cancer by dermal application on UV exposed mice. Int. J. Pharm..

[103] Kollipara R.K., Tallapaneni V., Sanapalli B.K.R., Vinoth Kumar G., Karri V.V.S.R. (2019). Curcumin loaded ethosomal vesicular drug delivery system for the treatment of melanoma skin cancer. Res. J. Pharm. Technol..

[104] Khan N.R., Wong T.W. (2018). 5-Fluorouracil ethosomes—Skin deposition and melanoma permeation synergism with microwave. Artif. Cells Nanomed. Biotechnol..

[105] Din F.U., Aman W., Ullah I., Qureshi O.S., Mustapha O., Shafique S., Zeb A. (2017). Effective use of nanocarriers as drug delivery systems for the treatment of selected tumors. Int. J. Nanomed..

[106] Lombardo D., Kiselev M.A., Caccamo M.T. (2019). Smart nanoparticles for drug delivery application: Development of versatile nanocarrier platforms in biotechnology and nanomedicine. J. Nanomater..

[107] Sunisha K., Kaushal P.M., Shyam B.S., Suman J. (2015). Ethosomes—A promising way for transdermal drug delivery. Int. J. Pharm. Sci. Res..

[108] Yingchoncharoen P., Kalinowski D.S., Richardson D.R. (2016). Lipid-based drug delivery systems in cancer therapy: What is available and what is yet to come. Pharmacol. Rev..

[109] Gharbavi M., Amani J., Kheiri-Manjili H., Danafar H., Sharafi A. (2018). Niosome: A promising nanocarrier for natural drug delivery through blood-brain barrier. Adv. Pharmacol. Sci..

[110] Mohamed M., Abu Lila A.S., Shimizu T., Alaaeldin E., Hussein A., Sarhan H.A., Szebeni J., Ishida T. (2019). PEGylated liposomes: Immunological responses. Sci. Technol. Adv. Mater..

[111] Jain S., Patel N., Shah M.K., Khatri P., Vora N. (2017). Recent advances in lipid-based vesicles and particulate carriers for topical and transdermal application. J. Pharm. Sci..

[112] Calienni M.N., Febres-Molina C., Llovera R.E., Zevallos-Delgado C., Tuttolomondo M.E., Paolino D., Fresta M., Barazorda-Ccahuana H.L., Gómez B., del Valle Alonso S. (2019). Nanoformulation for potential topical delivery of Vismodegib in skin cancer treatment. Int. J. Pharm..

